# Niosomes Functionalized with a Synthetic Carbohydrate Binding Agent for Mannose-Targeted Doxorubicin Delivery

**DOI:** 10.3390/pharmaceutics15010235

**Published:** 2023-01-10

**Authors:** Nastassja Burrini, Mario D’Ambrosio, Matteo Gentili, Roberta Giaquinto, Veronica Settimelli, Cristina Luceri, Marzia Cirri, Oscar Francesconi

**Affiliations:** 1 Department of Chemistry “Ugo Schiff” (DICUS), Università degli Studi di Firenze, Polo Scientifico e Tecnologico, 50019 Sesto Fiorentino, Italy; 2 Department of Neurosciences, Psychology, Drug Research and Child Health (NEUROFARBA), Section of Pharmacology and Toxicology, Università degli Studi di Firenze, Viale Pieraccini 6, 50139 Firenze, Italy

**Keywords:** synthetic receptors, nanoparticles, niosomes, functionalization, carbohydrate binding agent, drug delivery, cytotoxicity, doxorubicin, formulations

## Abstract

Niosomes are a potential tool for the development of active targeted drug delivery systems (DDS) for cancer therapy because of their excellent behaviour in encapsulating antitumorals and the possibility to easily functionalise their surface with targeting agents. Recently, some of us developed a synthetic carbohydrate binding agent (CBA) able to target the mannosidic residues of high-mannose-type glycans overexpressed on the surface of several cancer cell lines, promoting their apoptosis. In this article, we modified the structure of this mannose receptor to obtain an amphiphilic analogue suitable for the functionalization of doxorubicin-based niosomes. Several niosomal formulations and preparation methods were investigated deeply to finally obtain functionalized niosomes suitable for parental administration, which were stable for over six months and able to encapsulate up to 85% of doxorubicin (DOXO). In vitro studies, carried out towards triple-negative cancer cells (MDA-MB231), overexpressing high-mannose-type glycans, showed a cytotoxic activity comparable to that of DOXO but with an appreciable increment in apoptosis given by the CBA. Moreover, niosomal formulation was observed to reduce doxorubicin-induced cytotoxicity towards normal cell lines of rat cardiomyocytes (H9C2). This study is propaedeutic to further in vivo investigations that can aim to shed light on the antitumoral activity and pharmacokinetics of the developed active targeted DDS.

## 1. Introduction

Niosomes are self-assembled vesicular nanocarriers based on nonionic surfactants that have gained increasing scientific interest as versatile drug delivery systems (DDS) for several therapeutic applications [1]. The modulable chemical composition of their bilayer gives niosomes several advantages over liposomes, in terms of physical, chemical, and biological stability. Niosomes can encapsulate lipophilic, hydrophilic, and amphiphilic drugs and remain stable in the bloodstream for a reasonable time, which is useful for targeted drug delivery [2]. Niosomes have been successfully used to enhance the therapeutic efficacy of various drugs by increasing their bioavailability and reducing harmful side effects [3]. Because of these properties, they are particularly suitable for the development of active targeted DDS for cancer therapy, as many classical antitumoral drugs present a low selectivity for cancer cells and high toxicity towards healthy tissues [4,5,6]. Active targeting is usually achieved by functionalizing niosomes with a targeting agent that provides preferential accumulation of nanoparticles on the desired site of action [7,8]. This approach is based on specific interactions such as ligand–receptor, antibody–antigen, and lectin–carbohydrate [9]. Lectins are a class of nonimmunological proteins that selectively recognize and bind terminal oligosaccharides of glycoproteins and glycolipids expressed on the cell surfaces [10,11]. Different cell types express different glycans and changes in glycosylation are often a hallmark of a disease state. Therefore, DDS decorated with lectins of a certain carbohydrate selectivity can be used to target drugs to diseased cells depending on the carbohydrates exposed on their surfaces. In addition, lectin–carbohydrate binding mediates the endocytosis by target cells improving the cellular uptake of the drug [12,13]. Cancer cells frequently display glycans at different levels of expression or with fundamentally different structures than those observed on normal cells. For instance, high-mannose-type oligosaccharides are expressed on the surface of various cancer cells; however, they are not commonly found on the surface of normal cells [14]. Because mannose is prevalent as the terminal nonreductive sugar of glycans in cancer cells, terminal oligomannosides are attractive therapeutic targets. Recently, it was found that mannan-binding lectins themselves could demonstrate anticancer activity against animal model of human cancers, such as gastric [15], colon [16], and breast cancers [17], targeting the mannose residues on the cancer cell’s surface and inducing apoptosis and autophagy pathways in the malignant cells [18]. Plant lectins are mainly used for active targeted DDS; however, their development as therapeutics is hampered by their proteic nature. Indeed, lectins are expensive to produce, scale up, and purify and often present poor stability and problems of immunogenicity. In the last decades, small-size nonpeptidic carbohydrate binding agents (CBAs) have emerged as potential tools to overcome the limits of lectins [19,20]. Among nonpeptidic CBAs, one of the main classes is represented by biomimetic synthetic receptors for carbohydrates, which are developed to mimic the function of lectins in specific recognition events [21]. They are small molecules that exploit noncovalent interactions, used by their natural counterparts, to bind saccharides and feature some key advantages over lectins in terms of their higher stability and easier preparation, characterization, and structural modification.

**Figure 1:**
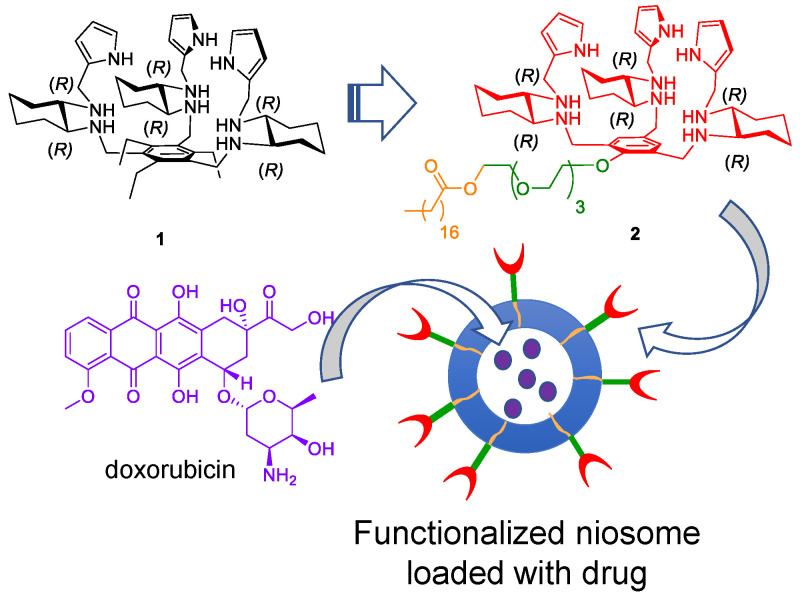
Concept of the work.

Recently, some of us reported a new set of biomimetic aminopyrrolic receptors that selectively bind mannose over other monosaccharides of biological interest [22,23,24,25]. Some of these compounds were found to possess antibiotic activity, in analogy to mannose-binding lectins, towards yeast and yeast-like microorganisms, an effect rationalized in terms of the ability to bind to cell-surface glycans, thereby interfering with key cellular processes [26]. They were also found to interact with the mannosylated glycoprotein gp120 of the HIV viral envelope, a glycoprotein crucial for the viral entry into the host cell, thereby preventing the HIV infection of CD4+ T-lymphocytes [27]. Moreover, investigation of the cytotoxic activity of these mannose-binding CBA showed that the one that has structure **1** (Figure 1), is able to induce a caspase-dependent apoptosis in HeLa, PLC/PRF/5, A549, and KG-1 cancer cell lines, with an extent of cytotoxicity (in the low micromolar range) directly related with the level of expression of high-mannose-type oligosaccharides on the surface of target cells [28]. In support of the mechanism of action, CBA **1** turned out to compete with Concanavalin A, a mannose-binding lectin, for the binding to high-mannose-type glycans on the cell surface of HeLa cells. Taking into consideration the previous results, CBA **1** might be a potential candidate for niosomes’ functionalization to develop active mannose-targeted DDS for nonselective anticancer drugs.

Among the nonselective antitumoral drugs, doxorubicin (DOXO) is one of the most widely used chemotherapeutic drugs for treating a myriad of cancers, including solid and metastatic tumours [29]. As well as its unique therapeutic properties, DOXO unfortunately presents severe problems of cardiotoxicity and induction of chemoresistance, two aspects that undermine its widespread applicability. Consequently, DOXO is a drug of choice for the development of active targeted DDS in order to reduce its side effects on normal cells [30].

Many attempts based on nanotechnology including liposomes, niosomes, polymeric micro- and nanoparticles, and glycosphingosomes [31,32] have been made in order to achieve a targeted drug delivery for DOXO aimed at promoting drug accumulation in specific sites, thus improving its therapeutic efficacy and minimizing its toxicity, by also exploiting PEGylation, magnetic drug targeting, folate-targeting systems, and modified mesoporous silica nanoparticles- or gold nanoparticles-based delivery systems [33,34,35,36,37].

We herein present the development of a mannose-targeted DDS consisting of niosomes loaded with DOXO and functionalized with a CBA for mannose. We report the design and synthesis of the new CBA **2** that shares the same binding pocket of CBA **1** but is endowed with an amphiphilic chain for niosomal functionalization, together with the preparation and characterization of the niosomal formulations. Finally, we describe the evaluation of the cytotoxic activity of prepared niosomes towards a triple-negative cancer cell line of breast cancer (MDA-MB231), known to overexpress high-mannose type glycans [38], and towards normal cell lines of rat cardiomyocytes (H9C2), particularly sensitive to the adverse effects of DOXO [39].

## 2. Materials and Methods

### 2.1. Synthesis of CBA ***2***

#### 2.1.1. Materials

Reagents were purchased from commercial suppliers and used without any further purification. 2-hydroxy-1,3,5-benzenetricarbaldehyde (**5**) was prepared according to known methods [40]. All moisture sensitive reactions were carried out in dried flasks under nitrogen atmosphere and using commercially available anhydrous solvents. Anhydrous tetrahydrofuran (THF) and Et_2_O were prepared by distillation over sodium and benzophenone. Dry dichloromethane (DCM) was kept over 3 Å molecular sieves. ^1^H-NMR and ^13^C-NMR spectra of characterization for compounds **2**–**4**, **6**–**9** are reported in Figures S1–S13 (as Supplementary Material).

#### 2.1.2. Compound **3**

Under nitrogen atmosphere, a mixture of stearic acid (10.0 g, 35.2 mmol) and SOCl_2_ (12 mL, 151 mmol) was refluxed for 30 min. The excess of SOCl_2_ was removed by bubbling nitrogen in the hot solution. The mixture was cooled to room temperature, the residue was dissolved in DCM (50 mL), and this solution was added dropwise in 1 h to a solution of tetraethylene glycol (68.4 g; 352 mmol) and Et_3_N (7.12 g; 70.4 mmol) under vigorous stirring. The mixture was stirred overnight at room temperature, then it was poured on water and extracted three times with DCM. The combined organic layers were washed three times with 1 M HCl, once with water, then dried over anhydrous Na_2_SO_4_ and concentrated to give 15.9 g of crude **3**, which was purified by flash column chromatography on silica gel (MeOH 20% in DCM) to afford pure **3** (14.6 g, 90%) as a white wax. ^1^H-NMR (200 MHz, CDCl_3_): δ 4.24–4.19 (m, 2H; CH_2_OC=O), 3.74–3.57 (m, 14H; CH_2_ tetraethylenic chain), 2.60 (bs, 1H; OH), 2.31 (m, 2H; CH_2_C=O), 1.63–1.56 (m, 2H; CH_2_CH_3_): 1.24 (s, 28H; CH_2_ alkyl chain), 0.89–0.83 ppm (m, 3H; CH_3_); ^13^C-NMR (50 MHz, CDCl_3_): δ 173.81, 72.50, 70.65, 70.55, 70.53, 70.35, 69.22, 63.29, 61.72, 34.18, 31.90, 29.67, 29.63, 29.59, 29.45, 29.33, 29.26, 29.12, 24.89, 22.66, 14.09 ppm.

#### 2.1.3. Compound **4**

Under nitrogen atmosphere, to a solution of **3** (1.01 g, 2.19 mmol) and Et_3_N (886 mg, 8.76 mmol) in toluene (5 mL) at 0 °C, methanesulfonyl chloride (276 mg; 2.41 mmol) was added, followed by the formation of a precipitate. The mixture was stirred for 2 h at room temperature, then the solvent was evaporated under reduced pressure and the residue was purified by flash column chromatography on silica gel (petroleum ether 30% in EA) to afford pure **4** (1.10 g, 93%) as a white wax. ^1^H-NMR (300 MHz, CDCl_3_): δ 4.40–4.37 (m, 2H; CH_2_OSO_2_CH_3_), 4.24–4.21 (m, 2H; CH_2_OC=O), 3.78–3.75 (m, 2H; CH_2_CH_2_OSO_2_CH_3_), 3.70–3.64 (m, 10H; CH_2_ tetraethylenic chain), 3.08 (s, 3H; OSO_2_CH_3_), 2.35–2.30 (m, 2H; CH_2_C=O): 1.64–1.56 (m, 2H; CH_2_CH_3_), 1.25 (s, 28H; CH_2_ alkyl chain), 0.90–0.85 ppm (m, 3H; CH_3_ alkyl chain); ^13^C-NMR (50 MHz, CDCl_3_): δ 173.86, 70.56, 69.22, 69.03, 63.28, 37.71, 34.20, 31.92, 29.69, 29.62, 29.48, 29.36, 29.29, 29.14, 24.91, 22.69, 14.13 ppm.

#### 2.1.4. Compound **6**

A mixture of **^5^** (7.60 g; 42.7 mmol), ethylene glycol (21.4 g; 344 mmol) and catalytic p-toluenesulfonic acid in toluene (100 mL), was refluxed overnight while removing water from the reaction with a Dean–Stark apparatus. The mixture was cooled to room temperature and washed three times with a saturated solution of NaHCO^3^, once with water, then dried over anhydrous Na^2^SO^4^ and concentrated to give crude **6** that was purified by flash column chromatography on silica gel (acetone 10% in DCM) to afford pure **6** (7.19 g, 88%) as a yellow wax. ^1^H-NMR (200 MHz, CDCl^3^): δ 8.35 (bs, 1H; OH), 7.49 (s, 2H; ArCH), 6.06 (s, 2H, OCHO ortho), 5.73 (s, 1H, OCHO para), 4.14–3.92 ppm (m, 12H; OCH^2^).

#### 2.1.5. Compound **7**

Under nitrogen atmosphere, to a solution of **5** (2.38 mg, 7.67 mmol) in dry DMF at 0 °C, NaH (291 mg, 60% in mineral oil, 7.28 mmol) was added. The mixture was stirred for 5 min, then it was allowed to warm to room temperature. Once the evolution of gas had completely stopped, **4** (4.13 g; 7.67 mmol) was added and the reaction was heated at 100 °C for 3 h. The solvent was evaporated under reduced pressure and the residue (6.50 g) was purified by flash column chromatography on silica gel (petroleum ether 30% in ethyl acetate) to afford pure **7** (5.71 g, 99%) as a white wax. ^1^H-NMR (300 MHz, CDCl_3_): δ 7.67 (s, 2H; Ar CH), 6.14 (s, 2H; ortho acetalic CH), 5.80 (s, 1H; para acetalic CH), 4.22–3.97 (m, 16H; CH_2_ acetal + CH_2_OC=O + CH_2_OAr), 3.82–3.79 (m, 2H), 3.73–3.63 (m, 10H; CH_2_ tetraethylenic chain): 2.33–2.28 (m, 2H; CH_2_C=O), 1.62–1.57 (m, 2H; CH_2_CH3), 1.24 (s, 28H; CH_2_ alkyl chain), 0.91–0.84 ppm (m, 3H; CH_3_ alkyl chain); ^13^C-NMR (50 MHz, CDCl3): δ 173.83, 157.05, 134.09, 131.48, 126.54, 103.17, 98.97, 75.83, 70.64, 70.57, 70.25, 69.20, 65.28, 65.16, 63.32, 34.19, 31.91, 29.68, 29.60, 29.46, 29.34, 29.27, 29.12, 24.89, 22.67, 14.11 ppm.

#### 2.1.6. Compound **8**

To a solution of **7** (1.09 mg, 1.45 mmol) in acetone (17 mL), HCl 6 M (3 mL) was added. The mixture was stirred for 20 min, then poured on water and extracted with DCM three times. The combined organic layers were washed three times with water, then dried over anhydrous Na_2_SO_4_ and concentrated to give 940 mg of crude 8, which was purified by flash column chromatography on silica gel (petroleum ether 30% in ethyl acetate) to afford pure **8** (800 mg, 89%) as an off-white wax. ^1^H-NMR (300 MHz, CDCl_3_): δ 10.47 (s, 2H; ortho CHO), 10.07 (s, 1H; para CHO), 8.58 (s, 2H; Ar CH), 4.46–4.43 (m, 2H; CH_2_OAr), 4.22–4.19 (m, 2H; CH_2_OC=O), 3.90–3.87 (m, 2H; CH_2_CH_2_OAr): 3.69–3.59 (m, 10H; tetraethylenic chain), 2.33–2.28 (m, 2H; CH_2_C=O), 2.28–1.60 (m, 2H; CH_2_CH_3_), 1.24 (s, 28H; CH_2_ alkyl chain), 0.89–0.85 (m, 3H; CH_3_ alkyl chain); ^13^C-NMR (50 MHz, CDCl_3_): δ 189.31, 188.20, 173.82, 167.51, 135.80, 132.49, 130.82, 78.60, 70.70, 70.56, 70.55, 70.55, 70.29, 69.17, 63.28, 34.19, 31.91, 29.68, 29.64, 29.60, 29.47, 29.35, 29.27, 29.14, 24.90, 22.68, 14.11 ppm.

#### 2.1.7. Compound **9**

To a solution of (1*R*,2*R*)-trans-1,2-diaminocyclohexane (5.45 g, 47.7 mmol) in CHCl_3_/MeOH 1:1 (80 mL), 1H-pyrrole-2-carboxaldehyde (454 mg; 4.77 mmol) was added and the solution was stirred overnight. NaBH_4_ (730 mg, 19.3 mmol) was added to the solution, followed by gas evolution. The mixture was stirred for 1.5 h at room temperature, then it was poured on water/brine 1:1 and extracted with CHCl3 three times. The combined organic layers were washed with water twice, dried over anhydrous Na_2_SO_4_, and concentrated to give 1.02 g of crude 9, which was purified by flash column chromatography on silica gel (MeOH 15% in CHCl_3_ + 1% NH_3_ sol. 33%) to afford pure **9** (868 mg, 94%) as a white solid. Excess of (1R,2R)-trans-1,2-diaminocyclohexane can be recovered by extraction with diethyl ether from aqueous phases after being saturated with KOH. m.p. = 70–71 °C; ^1^H-NMR (200 MHz, CDCl_3_): δ 9.48 (bs, 1H, pyrrolic NH), 6.71 (m, 1H, pyrrolic CH), 6.13–6.10 (m, 1H, pyrrolic CH), 6.00 (m, 1H, pyrrolic CH), 3.96 (d, J = 13.6 Hz, 1H; part A of an AB system, NHCH_2_pyr), 3.71 (d, J = 13.6 Hz, 1H; part B of an AB system, NHCH_2_pyr): 2.45–2.33 (m, 1H; CHNH), 2.18–2.06 (m, 2H); 1.92–1.60 (m, 6H); 1.39–0.88 ppm (m, 4H); ^13^C-NMR (50 MHz, CDCl_3_): δ 131.20, 116.91, 107.83, 105.57, 63.08, 55.28, 43.75, 35.83, 31.40, 25.21, 25.18 ppm.

#### 2.1.8. CBA **2**

A solution of **8** (353 mg, 0.416 mmol) and **9** (322 mg, 1.66 mmol) in CHCl_3_ (9 mL) was stirred for 40 min at room temperature. A freshly prepared suspension of NaBH_4_ (95 mg, 2.50 mmol) in MeOH (3 mL) was added under vigorous stirring, then the solution was stirred for 1 h at room temperature. The solution was poured on NaOH 1 M and extracted with CHCl_3_ three times. The organic layers were washed three times with water, dried over anhydrous Na_2_SO_4_, and concentrated to give 536 mg of crude **2** that was purified by flash column chromatography on silica gel (MeOH 10% in CHCl_3_ + 1% NH_3_ sol 33%) to afford pure **2** (334 mg, 70%) as a yellow oil. [α]D_23_ = −62.8 (CHCl_3_, c = 0.255); ^1^H-NMR (300 MHz, CDCl_3_): δ 9.66 (bs, 3H; NH pyrr), 7.19 (s, 2H; Ar CH), 6.61–6.59 (m, 3H; CH pyrr), 6.07–5.91 (m, 6H; CH pyrr), 4.22–4.18 (m, 2H; CH_2_OC=O), 4.05–3.54 (m, 26H): 2.33–2.06 (m, 20H), 1.72–1.58 (m, 8H), 1.26–0.86 ppm (m, 43H); ^13^C-NMR (75 MHz, CDCl_3_): δ 173.77, 154.39, 136.68, 133.74, 131.01, 130.73, 129.03, 117.16, 117.02, 107.72, 107.57, 106.06, 105.59, 73.46, 70.60, 70.55, 70.51, 69.20, 63.29, 60.73, 60.62, 50.01, 45.94, 43.51, 43.39, 34.19, 31.92, 31.46, 31.31, 29.68, 29.47, 29.33, 29.28, 29.14, 25.10, 24.99, 24.91, 22.68, 14.12 ppm. ESI-MS m/z (%): 1152.68 (100) [M + H]^+^, 577.18 (95) [M + 2H]^2+^; elemental analysis calcd. (%) for C_68_H_113_N_9_O_6_: C, 70.85; H, 9.88; N, 10.94; O, 8.33; found: C, 69.76; H, 9.90; N, 10.77.

### 2.2. Niosomes Preparation and Characterization

#### 2.2.1. Materials

Doxorubicin hydrochloride (DOXO) was purchased from Fluorochem Ltd. (Derbyshire, UK). Solulan C24 (Poly-24-oxyethylene cholesteryl ether, SOL) was kindly donated by Lubrizol (Cleveland, OH, USA). Cholesterol (CHL), sorbitan monostearate (Span 60), bovine serum albumin (BSA), chloroform (CHCl_3_), and acetonitrile (ACN) were provided by Merck KGaA (Darmstadt, Germany). Sodium lauryl sulphate (SLS) was from Galeno (Prato, Italy). Phosphate buffer solution (PBS, pH 7.4) was prepared according to Eur. Pharm. Purified water from Elix^®^ (Millipore, Burlington, MA, USA) was used. All other reagents were of analytical grade.

#### 2.2.2. Preparation of Empty Niosomes, Functionalized (NIO-F) or Nonfunctionalized (NIO)

Empty nonfunctionalized niosomal suspensions (NIO) were prepared by four different methods: Span 60, SOL, and CHL were used as vesicle forming agents in different molar ratios, starting from data reported in the literature [41] (see Table 1).

Thin layer evaporation–vortex (TLE-V) method: Span 60, CHL, and SOL were dissolved in CHCl_3_ in a round-bottom flask (organic phase); the solvent was removed by vacuum evaporation to obtain a thin layer, then hydrated with 20 mL PBS (pH 7.4). Three cycles of 3 min heating (65 °C) and 3 min vortexing (20 Hz) were performed.

Thin layer evaporation–paddle (TLE-P) stirring method: the thin layer obtained as above was hydrated with PBS at pH 7.4, stirred by a paddle at 700 rpm for 30 min at 65 °C.

Reverse phase evaporation method (REV-P): the surfactants–lipid mixture was dissolved in CHCl_3_, added to PBS and sonicated 3 h (Sonopuls HD 2200, Bandelin Electronic GmbH, Berlin, Germany) to form a W/O emulsion. CHCl_3_ was then removed by rotavapor (65 °C).

Chloroform injection: the organic phase (Span 60, CHL, and SOL dissolved in CHCl_3_) was injected into PBS by a syringe (flow rate 1.5 mL/min) under stirring (700 rpm). The organic solvent was then evaporated under vacuum (10 min, 35 °C).

To reduce the size, all the niosomal formulations were finally centrifuged 15 min at 4000 rpm (Hermle Z200A compact centrifuge, Labortechnik GmbH, Wehingen, Germany) and the supernatant was sonicated (Sonopuls HD 2200, 200 W power, probe KE 76, Bandelin Electronic GmbH, Berlin, Germany) for 2, 4, or 6 cycles of 5 min (50% of its maximum power).

Functionalized niosomes (NIO-F) were prepared by using two selected compositions: Span:CHL:SOL:Rec at 50:40:10:10 and 50:25:25:10 mol ratios, employing all the methods as described above. The CBA was solubilized in the organic phase together with the surfactants–lipid mixture. The surnatant obtained after centrifugation was sonicated for 6 cycles of 5 min.

#### 2.2.3. Preparation of Doxorubicin-Loaded Niosomes, Functionalized (NIO-F + DOXO) or Nonfunctionalized (NIO + DOXO)

Doxorubicin-loaded niosomes, both functionalized (NIO-F + DOXO) and nonfunctionalized (NIO + DOXO), were prepared according to the selected TLE-V and the REV-P methods at the selected composition Span 60:CHL:SOL(:Rec) 50:25:25(:10) molar ratios. The drug (2 mg/mL) was dissolved in the hydrophilic phase.

#### 2.2.4. Characterization of Niosomes

Niosomal formulations were characterized in terms of mean size, polydispersity index (PDI), and zeta (ζ) potential determined by DLS (Dynamic Light Scattering) using a ZetaSizer Pro Red-Label equipped with a ZS Explorer software (Malvern Panalytical Ltd., Malvern, UK) set at 25 ± 0.1 °C after suitable dilution with distilled water to prevent multiscattering phenomena.

The morphology of empty niosomes (NIO and NIO-F) was evaluated by Cryo Transmission Electron Microscope (Cryo-EM) (Thermo Scientific, Massachusetts, USA) with a X-FEG high-brightness gun 200 keV.

SEM analysis was performed on NIO-F + DOXO by using a FEI Quanta 200TM environmental scanning electron microscope (ESEM) (FEI Company, Oregon, USA), with a voltage of 30 kV.

Quantification of CBA **2** in niosomes was determined by UV-Vis spectroscopy and by 1H-NMR spectroscopy using phloroglucinol as internal standard. UV-Vis analysis was carried out on a known aliquot of lyophilized niosomes that was suspended in methanol and filtered on 0.2 µm PTFE filter. The obtained solution was analysed and concentration of **2** quantified (λ_max_ = 270 nm, ε = 1150 M^−1^ cm^−1^). 1H-NMR analysis was carried out on a known aliquot of lyophilized niosomes that was suspended in CD_3_OD and filtered on 0.2 µm PTFE filter. After addition of 15 µL of a 31.7 mM solution of phloroglucinol, concentration of CBA was determined by ^1^H-NMR by integration of pyrrolic signals of the CBA and aromatic singlet of phloroglucinol.

#### 2.2.5. Analytical Method for Doxorubicin Detection

The HPLC method for doxorubicin quantification was developed according to an opportunely modified method reported in the literature [39], by using a Merck Hitachi La Chrom Elite at λ = 480 nm. The column was an HIBAR RT RP-18e (150 mm × 4.6 mm, 5 μm). The mobile phase was acetonitrile (ACN): sodium laureth sulphate (SLS) 55:45 *v*/*v* at 1.2 mL/min flux, at an oven temperature of 40 °C. The volume injected was 20 µL and the retention time was 5.5 min. The drug chromatogram is reported in Figure S14 (as Supplementary material).

The linearity was determined on eight concentration levels ranging from 3.5 to 47.0 µg/mL (R^2^ = 0.997) with three injections for each level (LOQ = 0.27 µg/mL, LOD = 0.08 µg/mL).

#### 2.2.6. Drug Encapsulation Efficiency (%EE)

The %EE was indirectly determined after centrifugation (15 min, 5000 rpm) (HERMLE Labortechnik, mod. Z200A, Wehingen, Germany) of 1 mL of niosomal formulation placed in the upper chamber of a membrane concentrator (Vivaspin 2 MWCO 10,000, Vivascience AG, Hannover, Germany). The filtrate collected in the lower chamber was analysed for free drug determination by HPLC.

The % EE was determined according to the following equation:

(1) $$\%\mathrm{EE}\;=\frac{\mathrm{Ci}-\mathrm{Cf}}{\mathrm{Ci}}\times 100$$

where Ci is the initial drug concentration and Cf is the free drug concentration detected in the filtrate.

#### 2.2.7. Stability in Bovine Serum Albumin (BSA)

NIO-F + DOXO niosomes were incubated at 37 °C in a solution of BSA (40 mg/mL) in PBS pH 7.4 [42,43] Size, PDI and ζ-potential were monitored every 30 min over 12 h incubation.

#### 2.2.8. In Vitro Drug Release

Drug release studies from niosomal formulations were carried out according to the dialysis technique [44]. Experiments were carried out under sink conditions.

Briefly, 2 mL of niosomes dropped into a cellulose acetate dialysis bag (Spectra/Por^®^, MWCO 14,000, Spectrum, Mississauga, ON, Canada) were immersed in 150 mL of a PBS (pH 7.4) solution under stirring (75 rpm) at 37 °C for 24 h. Samples were withdrawn every 30 min for the first 5 h from the receiver solution, replaced with an equivalent volume of fresh PBS, and assayed by HPLC for drug concentration at 480 nm. The correction for the cumulative dilution was calculated. The release studies were performed in triplicate (C.V. < 3%) (Graph Pad Prism version 7.0 software (San Diego, CA, USA)).

#### 2.2.9. Stability Studies

Drug-loaded niosomal formulations were stored at 4 °C protected from light. Particle size, PDI, ζ-potential, and %EE were evaluated immediately on the freshly prepared formulations and monthly up to six months.

### 2.3. In Vitro Studies

#### 2.3.1. Cell-Culture Conditions

MDA-MB-231, human breast cancer cells, and H9C2, rat cardiomyoblasts (both from ATCC, Manassas, VA, USA), were maintained in Dulbecco’s modified Eagle’s medium (DMEM, Thermo Fisher Scientific, Rodano, Milan, Italy) supplemented with 10% fetal bovine serum (FBS, Thermo Fisher Scientific), 100 U/mL penicillin–streptomycin, 1% l-glutamine (200 mmol/L), at 37 °C in an atmosphere containing 5% CO_2_.

#### 2.3.2. Cell Viability Assay

MDA-MB-231 and H9C2 cells were seeded in 96-well plates, at a density of 5 × 10^3^ cells/well in 200 μL of medium. After 24 h incubation at 37 °C in 5% CO_2_, **1**, NIO-F, DOXO, NIO-F + DOXO, and NIO + DOXO were tested at concentrations ranging from 10^−3^ to 10^−9^ M and incubated for 72 h at 37 °C in 5% CO_2_. Cell viability was assessed by the colorimetric method based on [3-(4,5-dimethylthiazol-2-yl)-5-(3-carboxymethoxyphenyl)-2-(4-sulfophenyl)-2H-tetrazolium, inner salt; MTS] and an electron coupling reagent (phenazine ethosulfate; PES) (Promega Corporation, Madison, WI, USA). The optical density of the chromogenic product was measured at 490 nm. IC_50_ values were determined from survival curves calculated with GraphPad Prism 7 software (GraphPad, San Diego, CA, USA) [45].

#### 2.3.3. Apoptosis Determination

MDA-MB-231 cells were seeded on histological slides at a density of 1 × 10^5^ cells/well. After 24 h, cells were exposed to DOXO or to NIO + DOXO or to NIO-F + DOXO, at a final concentration of 10^−5^ M for 24 h. At the end of treatments, the cells were washed in PBS, fixed in Bouin’s liquid (acetic acid/formaldehyde/picric acid) for 20 min, and stained with Feulgen’s reaction. Briefly, cells were incubated in 1 N HCl for 22 min at 60 °C, with the Schiff reactive for 60 min at room temperature, and then washed in 0.05 N HCl and 5% NaHSO_3_. Successively, nuclei were counterstained for 30 s with 0.5% Fast Green in alcoholic solution, dehydrated in ethanol, washed in xylene, and mounted. Apoptotic cells were recognized by the presence of two morphological prodromal features of apoptotic bodies: nuclear fragmentation and cellular limits evanescence. The percentage of apoptotic cells was calculated on five microscopic fields for each experimental condition, with an average number of about 50 cells, and was determined by two independent observers, blindly [46].

## 3. Results and Discussion

### 3.1. Design and Synthesis of CBA

**Scheme 1:**
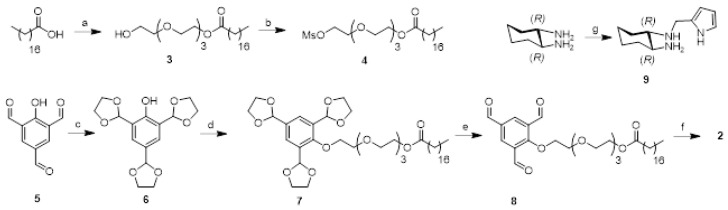
Synthesis of CBA **2**. Reagents: (a) SOCl_2_ then tetraethylene glycol, Et_3_N, DCM, 90%; (b) MsCl, Et_3_N, toluene, 93%; (c) ethylene glycol, p-TsOH cat., toluene, 88%; (d) 4, NaH, DMF, 99%; (e) HCl 6 M, acetone, 89%; (f) 9, CHCl_3_ then NaBH_4_, MeOH, 70%; (g) pyrrole-2-carboxaldehyde, CHCl_3_ then NaBH_4_, MeOH, 94%.

One of the most used strategies to functionalize niosomes with a targeting agent consists of modifying the agent’s structure by introducing an amphiphilic chain that can intercalate the niosomal bilayer with its lipophilic segment, thus protruding the hydrophilic portion, ending with the targeting agent, towards the aqueous bulk [2]. Although CBA **1** presents a hydrophilic binding site, in which the secondary amines are partially protonated at neutral pH, it presents only three short ethyl chains, unsuitable for niosome functionalization. Therefore, we modified the architecture of **1** to obtain CBA **2** (Figure 1) in which we preserved the hexaminotrypirrolic mannose-binding site while introducing, in place of the ethyl chains, a tetraethylene glycol monostearate tail. The amphiphilic chain was prepared (Scheme 1) by reacting stearyl chloride with an excess of tetraethylene glycol (TEG), affording the corresponding monoester derivative **3**, which was activated as the mesylate **4**. The trialdehyde **5**, which was prepared according to a known procedure [40], was protected as the triacetal **6** and O-alkylated with the mesylate **4** to give the intermediate **7**. After acetal deprotection of **7**, the aldehyde **8** was condensed with the (1R,2R)-*trans*-diaminocyclohexane derivative **9**, obtained from a reductive amination from (1R,2R)-*trans*-diaminocyclohexane and 1H-pyrrole-2-carboxaldehyde, and the resulting Schiff base was reduced in situ to the corresponding final hexamine **2**.

### 3.2. Preparation and Characterization of Empty Nonfunctionalized Niosomes (NIO)

In the first step of the work, NIO were prepared by four different preparation methods, thin layer evaporation–vortex (TLE-V), thin layer evaporation–paddle (TLE-P), reverse phase evaporation (REV-P), and chloroform injection (Chloroform inj.), in order to investigate the most suitable conditions to obtain niosomes for parenteral administration of DOXO. The target characteristics were a particle size ≤ 200 nm with a PDI ≤ 0.3 and a good stability in terms of ζ-potential. In general, a system may be considered stable when zeta potential values are around ±25 mV–±30 mV if only electrostatically stabilized. However, even lower zeta pot values are considered good if other stabilization effects also occur (i.e., steric effects) [47].

The composition consisting of Span 60:CHL:SOL in the 50:40:10 molar ratio, reported in the literature [39], was taken as the starting point from which the concentrations of the components were varied (see Table 1) to investigate their effect on the niosomal characteristics. Span 60 was used as the principal surfactant, CHL as the stabilizer due to its effect to impart rigidity to niosomes, and SOL as the emulsifier due to its ability to improve the membrane fluidity and elasticity leading to homogenous spherical vesicles [48] and its steric stabilization effect preventing vesicle aggregation [49].

Starting from the Span 60:CHL:SOL 50:40:10 composition the Span 60:CHL ratio was inverted (40:50:10) and then the amount of SOL was increased from 10 to 25% mol, in both the two formulations, by removing the same amount of Span 60 (35:40:25 and 25:50:25) or CHL (50:25:25 and 40:35:25). All the formulations were initially subjected to two sonication cycles of 5′. The results in terms of mean size, PDI, and ζ-potential of all the formulations are collected in Table 1. All the tested methods except chloroform injection were able to produce vesicles with the required size at the starting composition 50:40:10, whereas a trend to size increase was noted when inverting the Span 60:CHL ratio from 50:40:10 to 40:50:10, regardless of the preparation technique. In any case, a similar trend was also observed when CHL was in a higher molar ratio respect to Span 60, giving rise to the size exceeding 200 nm in most cases. Both PDI and ζ-potential resulted as being suitable in all cases.

The Span 60:CHL:SOL 50:40:10 formulations obtained by the TLE-V, TLE-P, and REV-P methods were then selected, meeting the desired properties for parenteral administration. Since the only formulation prepared by the chloroform injection method falling within the desired range was that of 50:25:25, this one was selected for the other methods in addition to the formulation 50:40:10 in order to further investigate the influence of all the preparation methods in the subsequent preparation of functionalized niosomes.

Before passing to the functionalization phase, the selected formulations were subjected to different cycles of sonication, to evaluate the possible effect of such a process parameter on the size of the vesicle. The number of sonication cycles was increased from to two to four and six. The sonication did not significantly affect the size for all the tested preparation techniques except for the TLE-V method where the treatment with six sonication cycles for 5 min allowed a further decrease in the average diameter of the niosomes. Probably, niosomes with an initial lower size range showed a smaller degree of size reduction than the other formulations due to their higher thermodynamic stability, leading to equilibrium being achieved quickly with a minimal size reduction [50]. All the ζ-potential values fell in the acceptable range for stability. The results in terms of size, PDI, and ζ-potential of the selected formulations sonicated for six cycles of 5′ are summarized in Table 2.

### 3.3. Preparation and Characterization of Empty Functionalized Niosomes (NIO-F)

The selected formulations were then functionalized with the CBA (with compositions 50:40:10:10 and 50:25:25:10 mol) and characterized to evaluate the effects of functionalization on their dimensions. The functionalization of niosomes led to an increase in the size of vesicles with composition 50:40:10:10 regardless of the preparation method, achieving values higher than 200 nm, the maximum value acceptable for an *i.v.* administration. Such an effect could probably be ascribed to the lower concentration of the surfactant SOL. Analogous results were also obtained for the 50:25:25:10 formulations prepared by the TLE-P method, maybe due to the different energy supplied by the different methods. Thus, such formulations were discarded. Conversely, the niosomal formulations 50:25:25:10 obtained by the other three methods, despite showing a slight increase, maintained the size values within the desired range and with suitable values of ζ-potential (138.0 nm, −16.3 mV for TLE-V, 161.9 nm, −11.7 mV for REV-P, 143.9 nm, −14.1 mV for Chloroform Inj.) with PDI values of 0.2, indicating homogeneous systems. Therefore, these formulations were further characterized in terms of CBA concentration. The formulation prepared by the chloroform injection method showed a limited CBA concentration, much lower than the corresponding formulations obtained by the REV-P and TLE-V methods (0.085 mM vs. 0.511 mM of REV-P and 0.580 mM of TLE-V). Despite the dimensions falling in the required range, this method was also then discarded.

**Figure 2:**
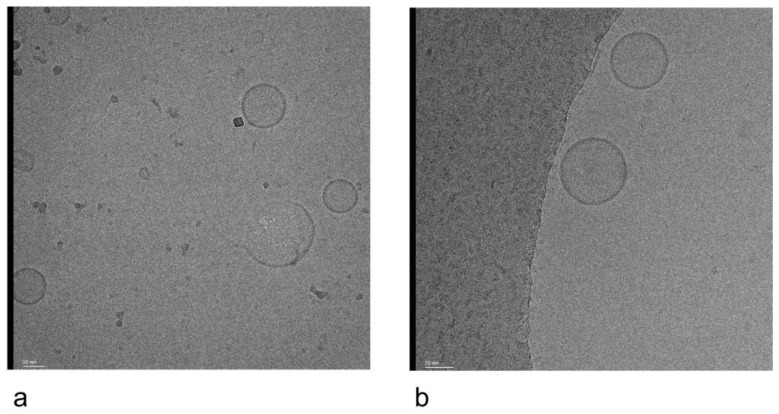
CRYO-EM images of NIO-F prepared by TLE-V (**a**) and REV-P method (**b**).

Niosomes prepared by the TLE-V and REV-P methods were also characterized by Cryo-EM that allows visualization of samples in their native frozen-hydrated state (Figure 2). Cryo-EM analysis revealed the presence of single nanovesicles with a spherical shape and a dimensional range consistent with the size distribution observed by DLS, showing smaller vesicles for the TLE-V compared to the REV-P method.

Based on these results, the 50:25:25 niosomal formulations, both NIO-F (50:25:25:10) or NIO (50:25:25), prepared by TLE-V and REV-P were then selected for drug loading. DOXO was added in the aqueous phase at a concentration of 2 mg/mL (50:25:25:10:10 and 50:25:25:10).

### 3.4. Preparation and Characterization of Drug-Loaded Niosomes, Functionalized (NIO-F + DOXO) or Nonfunctionalized (NIO + DOXO)

The drug loading led to a slight increase in the dimensions without affecting them significantly for both NIO + DOXO or NIO-F + DOXO niosomes. In the case of functionalized niosomes, after DOXO-loading, the ζ-potential tended to achieve more positive values, maybe due to the positively charged hydrophilic drug.

**Figure 3:**
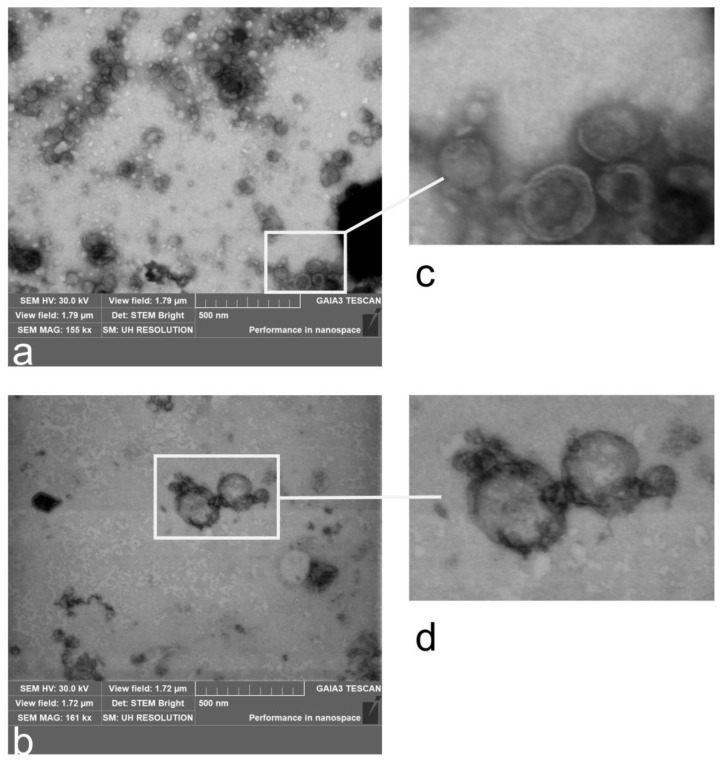
SEM micrographs of NIO-F + DOXO obtained by TLE-V (**a**) and REV-P method (**b**) and their magnification (**c**,**d**).

SEM analysis (Figure 3) was performed on functionalized DOXO-loaded niosomes prepared by TLE-V (Figure 3a) and REV-P (Figure 3b) to investigate their morphology.

Spherical nanovesicles were obtained by both the TLE-V and REV-P methods, differing for their structure. As can be observed by magnification, niosomes prepared by the TLE-V method appeared as Multi-Lamellar Vesicles (MLV) (Figure 3c), while Large Unilamellar Vesicles (LUV) were obtained by the Rev-P method (Figure 3d).

The size, PDI, and ζ-potential of all the drug-loaded formulations were monitored for up to two weeks to select the best formulations.

In the case of nonfunctionalized niosomes, the characteristics remained optimal for both formulations, regardless of the preparation method. Conversely, a significant increase in the size, exceeding 200 nm, was observed in the case of functionalized niosomes prepared with the REV-P method, thus resulting in them not being suitable for intravenous administration. The PDI also increased reaching values over 0.4, thus indicating a nonhomogeneous system (Table 3).

For this reason, the drug-loaded functionalized niosomal formulation obtained by REV-P was discarded, while that obtained by the TLE-V method was selected for further studies and compared to the corresponding formulation of NIO + DOXO.

Functionalized niosomes allowed a higher %EE than the nonfunctionalized ones, reaching values of 84.7% vs. 40% of nonfunctionalized niosomes, maybe due to the change in the physico-chemical properties of the niosomal membrane leading to nonspecific interactions in the presence of the CBA.

**Figure 4:**
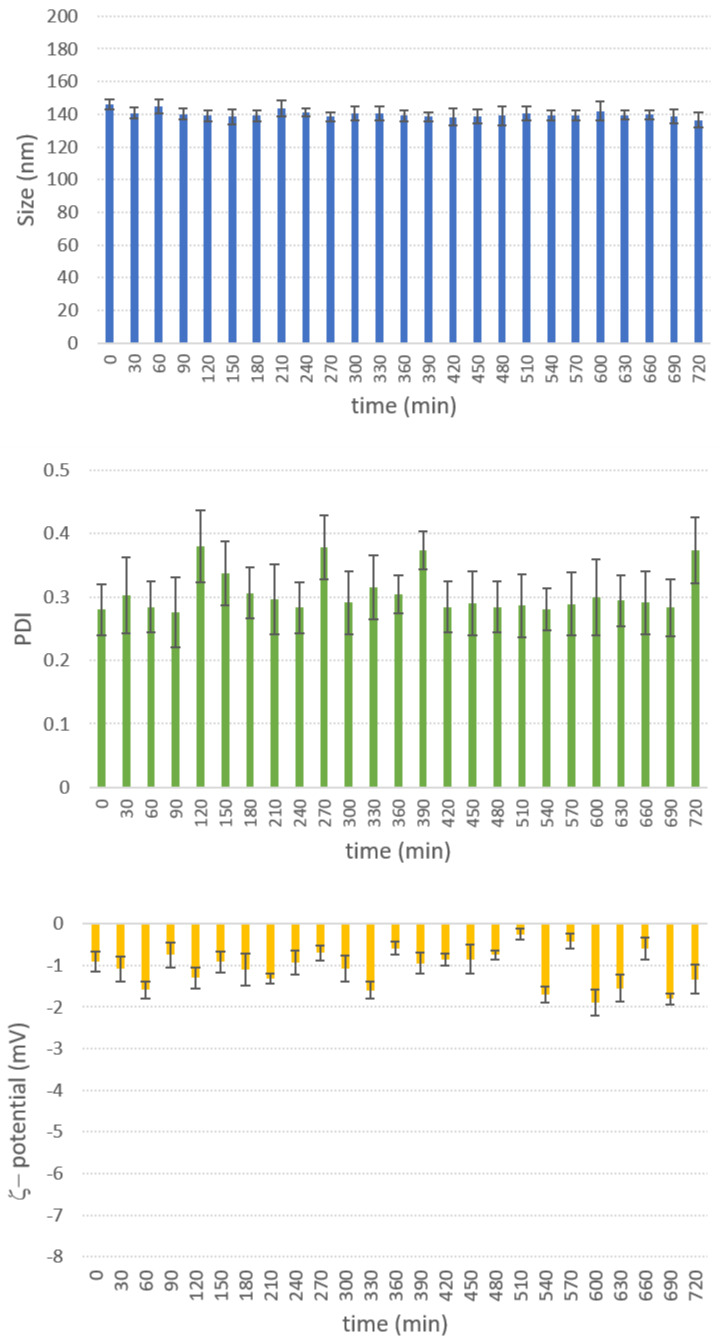
Stability studies of NIO-F + DOXO in a solution of BSA in PBS over 12 h in terms of size, PDI, and ζ-potential.

The stability of NIO-F + DOXO was verified by incubating them in a solution of bovine serum albumin (BSA) in phosphate buffer saline (PBS, pH = 7.4) at 37 °C to mimic the physiological conditions of parenterally administered preparations. The size, the PDI, and the ζ-potential were monitored every 30′ over 12 h. The mean size remained constant, only showing slight but not significant variations (*p* > 0.05) in the PDI values, thus indicating the stability of the developed niosomes in BSA (Figure 4). Similar values were registered after 24 h (138.7 nm with a PDI value of 0.34 and ζ-potential of −1.5 mV).

In vitro release studies of DOXO from the NIO-F + DOXO were performed according to the dialysis bag method at 37 °C up to 24 h by monitoring the drug release every 30 min for the first 5 h and compared to the corresponding nonfunctionalized formulation. The release profiles showed a similar behaviour, but the functionalization led to the improvement in the drug release properties, maybe due to possible changes in the bilayer structure properties of niosomes in the presence of the CBA.

**Figure 5:**
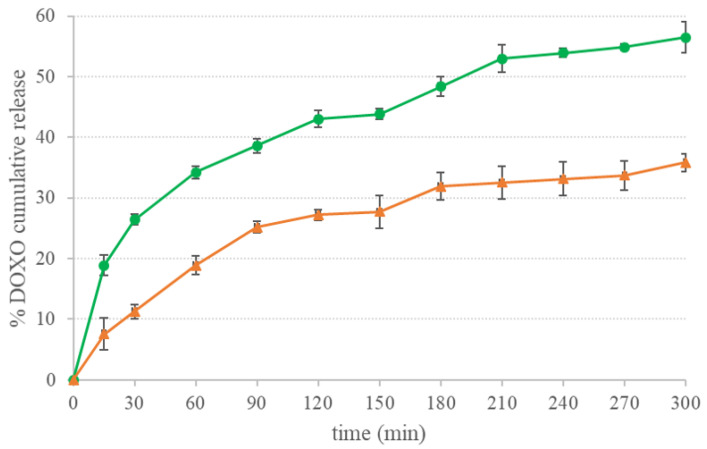
DOXO release profiles from niosomal formulations: (▲) NIO + DOXO and (●) NIO-F + DOXO.

In the case of NIO + DOXO, the drug release reached values of about 25% in the first 120 min and then slowly increased not exceeding values of 35% after 5 h. In the case of NIO + DOXO, just in the first 120 min, the release of the drug exceeded 40% followed by a gradually increasing phase achieving values near 60% after 5 h (Figure 5). Both niosome formulations showed a sustained drug release up to 24 h, maintaining the same trend, reaching values of 55.4% doxorubicin release from NIO + DOXO and 85.7% from NIO-F + DOXO, respectively.

The stability of the NIO-F + DOXO was evaluated over time both in terms of size, PDI, ζ-pot, and %EE. One of the major problems niosomes can meet over time is the leakage or the loss of the drug from the niosomal system. The formulations were stocked in glass vials at 4 °C.

The data obtained in terms of size, PDI, and ζ-pot, collected in Table 4, indicated that NIO-F + DOXO were stable up to 6 months: the size and PDI remained almost unchanged, while the ζ-potential showed an increase after 2 months of storage. The %EE did not fall below 80% after 6 months, thus indicating the stability of the developed formulation, ruling out any drug leakage process.

### 3.5. Cell Viability Assay

**Figure 6:**
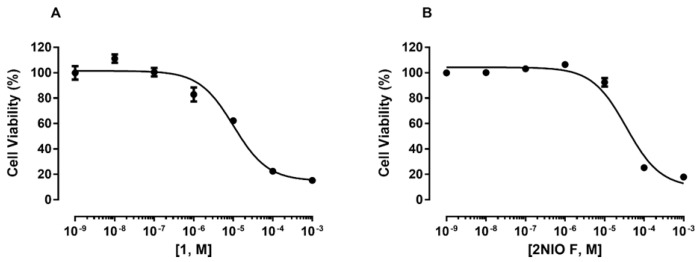
Cell viability of MDA-MB-231 exposed to various concentrations of CBA **1** (**A**) or to functionalized niosomes (NIO-F) where concentrations are expressed in moles of CBA **2** (2NIO-F) (**B**), for 72 h. Data are expressed as means ± SE.

Cell viability was measured in MDA-MB-231 cells exposed for 72 h to CBA **1** or to NIO-F prepared with the amphiphilic analogue **2**. CBA **1** inhibited breast cancer cell viability with an IC_50_ value of 1.02 × 10^−5^ M, and this effect was maintained when using NIO-F with analogue **2**, IC_50_ of 3.57 × 10^−5^ M (Figure 6). Gratifyingly, this behaviour clearly indicates that the structural modifications carried out on **1** to obtain **2** do not affect the cytotoxic ability, and, moreover, that CBA **2** assembled in the niosomal formulation is correctly presented to MDA-MB-231 cells to be explicit in its activity. Given that the cytotoxic activity of CBA **1** is triggered by mannose recognition on the surface of cancer cells [28], comparable cytotoxicity observed for CBA **2** strongly suggests that mannose recognition, an essential feature for a mannose-targeted DDS, occurs as well when the CBA is assembled on the niosomal surface.

**Figure 7:**
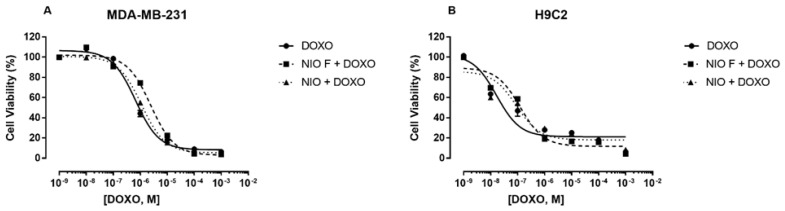
Effect of DOXO, NIO-F + DOXO, and NIO + DOXO, on MDA-MB-231 (**A**) and H9C2 (**B**) cells’ viability after 72 h of exposure. Data are expressed as means ± SE.

Cell viability was than measured in MDA-MB-231 and H9C2 cells using DOXO and the DOXO-loaded niosomal formulation (NIO-F + DOXO and NIO + DOXO). As expected, the cytotoxicity of DOXO was higher in normal H9C2 cells than in MDA-MB-231 breast cancer cells (IC_50_ of 1.68 × 10^−8^ vs. 6.65 × 10^−7^ M) (Figure 7). NIO-F + DOXO and NIO + DOXO had similar effects to DOXO on cell viability in MDA-MB-231 cells, with IC_50_ values of 2.44 × 10^−6^ and 1.12 × 10^−6^ M, respectively. Conversely, on normal H9C2 cells, we observed a strong increase in IC_50_ values for both niosomal systems (IC_50_ of 1.25 × 10^−7^ and 8.99 × 10^−8^ M, respectively). Unsurprisingly, with the cytotoxicity of DOXO being from 2 to 3 orders of magnitude higher than that of CBA **2**, the contribution to cytotoxicity given by the functionalization is not observable. However, the results highlight the role of the niosomal formulation in protecting normal cells from the cytotoxic effect of DOXO, which instead remains preserved towards cancer cells.

### 3.6. Apoptosis Determination

**Figure 8:**
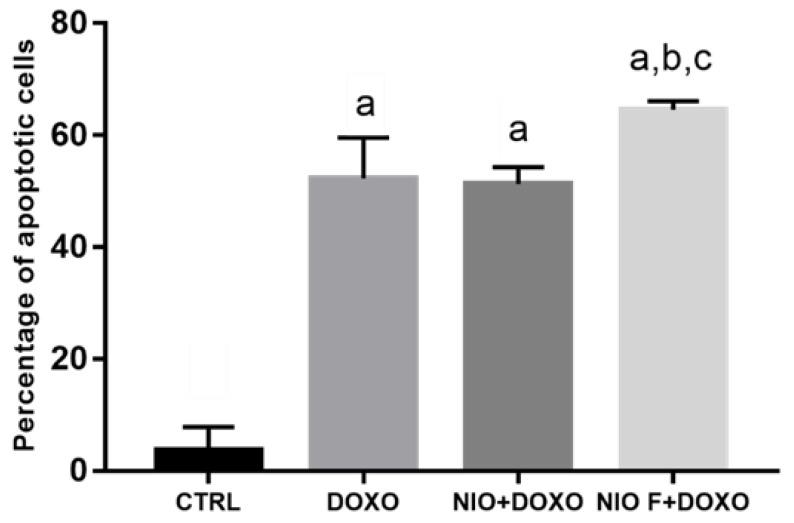
Percentage of apoptotic cells in MDA-MB-231 cells exposed to DOXO, NIO + DOXO, or NIO-F + DOXO, 10^−5^ M for 24 h. Data are expressed as mean ± S E. a = *p* < 0.001 vs. CTRL; b = *p* < 0.01 vs. NIO, c = *p* < 0.05 vs. DOXO by ANOVA and Tukey’s multiple comparisons test.

Feulgen staining was used to identify apoptotic cells. A significant increase in the number of apoptotic cells was observed in all treated cells compared to the control ones. MDA- MB-231 cells exposed to NIO-F + DOXO exhibited a significantly higher percentage of apoptotic cells compared to those treated with NIO + DOXO (*p* < 0.01) or with DOXO (*p* < 0.05) (Figure 8). Despite the prominent role of DOXO, CBA **2** decorating the functionalized niosomes actively contributed to the promotion of apoptosis of cancer cells, following the behaviour observed for its progenitor **1** [28].

## 4. Conclusions

In this article, we demonstrated the possibility of using a synthetic CBA to functionalize doxorubicin-loaded niosomes for the development of a mannose-targeted DDS. Through the structural modification of a previously presented CBA, known to target high-mannose-type glycans on the surface of cancer cells inducing apoptosis, we synthesized an amphiphilic analogue for niosome functionalization. With this analogue, we developed doxorubicin-loaded functionalized niosomes suitable for parental administration, able to reach an encapsulation efficacy of 85%, ensuring a controlled release and showing good stability. In vitro studies carried out towards MDA-MB-231 showed a cytotoxic activity for CBA **2** comparable with its progenitor **1**, which confirms the correct presentation of CBA **2** on niosomes. Moreover, DOXO-loaded functionalized niosomes showed not only comparable cytotoxicity to DOXO, but also an appreciable increment in apoptosis provided by CBA. Finally, comparison studies carried out with normal H9C2 cells showed a protective role of niosomal formulation on cardiomyocytes. These encouraging results open the way to further in vivo studies that can aim to shed light on the antitumoral activity and pharmacokinetics of this mannose-targeted DDS.

## Figures and Tables

**Table 1:** Effect of different preparation methods: thin layer evaporation–vortex (TLE-V), thin layer evaporation–paddle (TLE-P), reverse phase evaporation (REV-P), and chloroform injection (Chloroform inj.) on size, polydispersion index (PDI), and ζ-potential of NIO.

Method	Span:CHL:SOL (% mol)	Mean Size (nm) ± S.D	PDI ±S.D	ζ-Pot (mV) ± S.D
TLE-V	50:40:10	182.2 ± 3.1	0.21 ± 0.01	−37.8 ± 2.7
TLE-V	40:50:10	197.4 ± 1.0	0.29 ± 0.04	−39.4 ± 2.3
TLE-V	35:40:25	252.0 ± 2.9	0.20 ± 0.02	−15.7 ± 1.0
TLE-V	50:25:25	216.2 ± 7.1	0.18 ± 0.01	−19.5 ± 2.6
TLE-V	25:50:25	198.8 ± 3.2	0.25 ± 0.02	−17.4 ± 1.2
TLE-V	40:35:25	183.0 ± 1.6	0.22 ± 0.01	−19.9 ± 1.6
TLE-P	50:40:10	161.2 ± 0.8	0.18 ± 0.01	−27.3± 2.6
TLE-P	40:50:10	191.2 ± 4.1	0.17 ± 0.01	−27.0 ± 1.8
TLE-P	35:40:25	214.5 ± 1.4	0.21 ±0.01	−15.8 ± 1.7
TLE-P	50:25:25	186.6 ± 1.9	0.20 ± 0.01	−18.4 ± 2.7
TLE-P	25:50:25	250.4 ± 3.4	0.19 ± 0.02	−15.1 ± 1.1
TLE-P	40:35:25	189.1 ± 1.1	0.18 ± 0.03	−17.6 ± 1.7
REV-P	50:40:10	160.6 ± 1.5	0.21 ± 0.01	−25.7 ± 1.7
REV-P	40:50:10	268.7 ± 2.5	0.22 ± 0.02	−28.3 ± 1.4
REV-P	35:40:25	187.6 ± 1.4	0.22 ± 0.01	−16.6 ± 1.3
REV-P	50:25:25	123.9 ± 0.4	0.19 ± 0.01	−14.5 ± 0.8
REV-P	25:50:25	195.3 ± 3.5	0.22 ± 0.01	−18.9 ± 1.2
REV-P	40:35:25	172.8 ± 0.1	0.19 ± 0.02	−14.0 ± 1.1
Chloroform inj.	50:40:10	257.9 ± 2.4	0.27 ± 0.01	−14.8 ± 0.5
Chloroform inj.	40:50:10	320.9 ± 6.0	0.40 ± 0.05	−15.9 ± 0.8
Chloroform inj.	35:40:25	288.2 ± 2.4	0.23 ± 0.01	−11.6 ± 0.1
Chloroform inj.	50:25:25	134.7 ± 1.6	0.21 ± 0.01	−17.6 ± 2.3
Chloroform inj.	25:50:25	254.0 ± 1.3	0.26 ± 0.01	−16.5 ± 1.9
Chloroform inj.	40:35:25	205.1 ± 1.6	0.16 ± 0.01	−15.5 ± 1.7

**Table 2:** Mean size, polydispersion index (PDI), and ζ-potential of empty niosomes sonicated for 6 cycles of 5′ selected for the subsequent functionalization.

Preparation Method	Span60:CHL:SOL (% mol)	Mean Size (nm) ± S.D	PDI ±S.D	ζ-Potential (mV) ± S.D
TLE-V	50:40:10	159.4 ± 1.2	0.16 ± 0.01	−35.3 ± 8.3
TLE-V	50:25:25	127.5 ± 2.3	0.16 ± 0.01	−18.3 ± 3.0
TLE-P	50:40:10	152.4± 0.9	0.15 ± 0.01	−25.9 ± 4.8
TLE-P	50:25:25	189.7 ± 2.4	0.21 ± 0.02	−18.1 ± 2.2
REV-P	50:40:10	150.2 ± 2.0	0.15 ± 0.01	−24.2 ± 1.9
REV-P	50:25:25	125.5 ± 0.3	0.18 ± 0.01	−12.6 ± 1.4
Chloroform inj.	50:25:25	135.0 ± 0.3	0.18 ± 0.01	−12.6 ± 1.5

**Table 3:** Stability of NIO-F + DOXO prepared by TLE-V (84.7% EE) and REV-P (89.5% EE) methods over 2 weeks in terms of size, PDI, and ζ-potential.

	TLE-V Niosomes			REV-P Niosomes		
	Mean Size (nm) ±S.D.	PDI ± S.D.	ζ-Pot (mV) ± S.D.	Mean Size (nm) ±S.D.	PDI ± S.D.	ζ-Pot (mV) ±S.D.
Freshly prepared	147.3 ± 1.4	0.28 ± 0.04	−0.91 ± 0.24	170.2 ± 0.8	0.23 ± 0.02	−2.15 ± 0.01
After 24 h	140.1 ± 0.2	0.24 ± 0.01	0.95 ± 0.02	267.0 ± 0.5	0.51 ± 0.08	−0.10 ± 0.15
After 72 h	134.1 ± 1.3	0.20 ± 0.11	1.66 ± 0.18	307.7 ± 0.1	0.45 ± 0.01	−1.87 ± 0.65
After 2 weeks	170.0 ± 5.6	0.34 ± 0.03	6.34 ± 0.55	315.6 ± 4.6	0.50 ± 0.03	−3.10 ± 0.55

**Table 4:** Stability studies of NIO-F + DOXO over 6 months.

NIO-F + DOXO	Mean Size (nm) ± S.D.	PDI ± S.D.	ζ-Pot (mV) ± S.D.	%EE
Freshly prepared	147.3 ± 1.4	0.28 ± 0.04	−0.91 ± 0.24	84.7 ± 2.8
1 month	160.0 ± 12.5	0.25 ± 0.01	−4.52 ± 0.33	84.1 ± 1.6
2 months	141.0 ± 3.2	0.25 ± 0.01	−0.85 ± 0.65	83.5 ± 2.0
4 months	138.6 ± 1.7	0.22 ± 0.01	24.91 ± 0.94	82.1 ± 1.8
6 months	122.0 ± 2.6	0.18 ± 0.01	30.88 ± 0.37	81.0 ± 1.5

## Data Availability

Not applicable.

## Supplementary Materials

The following supporting information can be downloaded at: https://www.mdpi.com/article/10.3390/pharmaceutics15010235/s1, Figure S1: ^1^H NMR spectrum of compound **3** (200 MHz, CDCl_3_); Figure S2: ^13^C NMR spectrum of compound **3** (50 MHz, CDCl_3_); Figure S3: ^1^H NMR spectrum of compound **4** (300 MHz, CDCl_3_); Figure S4: ^13^C NMR spectrum of compound **4** (50 MHz, CDCl_3_); Figure S5: ^1^H NMR spectrum of compound **6** (200 MHz, CDCl_3_); Figure S6: ^1^H NMR spectrum of compound **7** (300 MHz, CDCl_3_); Figure S7: ^13^C NMR spectrum of compound **7** (50 MHz, CDCl_3_); Figure S8: ^1^H NMR spectrum of compound **8** (300 MHz, CDCl_3_); Figure S9: ^13^C NMR spectrum of compound **8** (50 MHz, CDCl_3_); Figure S10: ^1^H NMR spectrum of compound **9** (200 MHz, CDCl_3_); Figure S11: ^13^C NMR spectrum of compound **9** (50 MHz, CDCl_3_); Figure S12: ^1^H NMR spectrum of receptor **2** (300 MHz, CDCl_3_); Figure S13: ^13^C NMR spectrum of receptor **2** (75 MHz, CDCl_3_); Figure S14: HPLC chromatogram of DOXO.

## References

[1] Khoee S., Yaghoobian M. (2017). Niosomes: A Novel Approach in Modern Drug Delivery Systems. Nanostructures for Drug Delivery.

[2] Momekova D.B., Gugleva V.E., Petrov P.D. (2021). Nanoarchitectonics of Multifunctional Niosomes for Advanced Drug Delivery. ACS Omega.

[3] Muzzalupo R., Mazzotta E. (2019). Do Niosomes Have a Place in the Field of Drug Delivery?. Expert Opin. Drug Deliv..

[4] Argenziano M., Arpicco S., Brusa P., Cavalli R., Chirio D., Dosio F., Gallarate M., Peira E., Stella B., Ugazio E. (2021). Developing Actively Targeted Nanoparticles to Fight Cancer: Focus on Italian Research. Pharmaceutics.

[5] Aparajay P., Dev A. (2022). Functionalized Niosomes as a Smart Delivery Device in Cancer and Fungal Infection. Eur. J. Pharm. Sci..

[6] Böttger R., Pauli G., Chao P.-H., al Fayez N., Hohenwarter L., Li S.-D. (2020). Lipid-Based Nanoparticle Technologies for Liver Targeting. Adv. Drug Deliv. Rev..

[7] Kim G.J., Nie S. (2005). Targeted Cancer Nanotherapy. Mater. Today.

[8] Tavano L., Muzzalupo R. (2016). Multi-Functional Vesicles for Cancer Therapy: The Ultimate Magic Bullet. Colloids Surf B Biointerfaces.

[9] Mager M.D., LaPointe V., Stevens M.M. (2011). Exploring and Exploiting Chemistry at the Cell Surface. Nat. Chem..

[10] Ghazarian H., Idoni B., Oppenheimer S.B. (2011). A Glycobiology Review: Carbohydrates, Lectins and Implications in Cancer Therapeutics. Acta Histochem..

[11] Dube D.H., Bertozzi C.R. (2005). Glycans in Cancer and Inflammation—Potential for Therapeutics and Diagnostics. Nat. Rev. Drug Discov..

[12] Zhang R., Qin X., Kong F., Chen P., Pan G. (2019). Improving Cellular Uptake of Therapeutic Entities through Interaction with Components of Cell Membrane. Drug Deliv..

[13] Gabor F., Bogner E., Weissenboeck A., Wirth M. (2004). The Lectin–Cell Interaction and Its Implications to Intestinal Lectin-Mediated Drug Delivery. Adv. Drug Deliv. Rev..

[14] De Leoz M.L.A., Young L.J.T., An H.J., Kronewitter S.R., Kim J., Miyamoto S., Borowsky A.D., Chew H.K., Lebrilla C.B. (2011). High-Mannose Glycans Are Elevated during Breast Cancer Progression. Mol. Cell. Proteom..

[15] Sato Y., Kubo T., Morimoto K., Yanagihara K., Seyama T. (2016). High Mannose-Binding Pseudomonas Fluorescens Lectin (PFL) Downregulates Cell Surface Integrin/EGFR and Induces Autophagy in Gastric Cancer Cells. BMC Cancer.

[16] Minko T. (2004). Drug Targeting to the Colon with Lectins and Neoglycoconjugates. Adv. Drug Deliv. Rev..

[17] Sindhura B.R., Hegde P., Chachadi V.B., Inamdar S.R., Swamy B.M. (2017). High Mannose N-Glycan Binding Lectin from Remusatia Vivipara (RVL) Limits Cell Growth, Motility and Invasiveness of Human Breast Cancer Cells. Biomed. Pharmacother..

[18] Mazalovska M., Kouokam J.C. (2020). Plant-Derived Lectins as Potential Cancer Therapeutics and Diagnostic Tools. Biomed Res. Int..

[19] Balzarini J. (2007). Targeting the Glycans of Glycoproteins: A Novel Paradigm for Antiviral Therapy. Nat. Rev. Microbiol..

[20] Francesconi O., Roelens S. (2019). Biomimetic Carbohydrate-Binding Agents (CBAs): Binding Affinities and Biological Activities. ChemBioChem.

[21] Gentili M., Nativi C., Francesconi O., Roelens S. (2016). Synthetic Receptors for Molecular Recognition of Carbohydrates. Carbohydrate Chemistry.

[22] Nativi C., Francesconi O., Gabrielli G., Vacca A., Roelens S. (2011). Chiral Diaminopyrrolic Receptors for Selective Recognition of Mannosides, Part 1: Design, Synthesis, and Affinities of Second-Generation Tripodal Receptors. Chem.-A Eur. J..

[23] Ardá A., Venturi C., Nativi C., Francesconi O., Gabrielli G., Cañada F., Jiménez-Barbero J., Roelens S. (2010). A Chiral Pyrrolic Tripodal Receptor Enantioselectively Recognizes β-Mannose and β-Mannosides. Chem.-A Eur. J..

[24] Ardá A., Cañada F.J., Nativi C., Francesconi O., Gabrielli G., Ienco A., Jiménez-Barbero J., Roelens S. (2011). Chiral Diaminopyrrolic Receptors for Selective Recognition of Mannosides, Part 2: A 3D View of the Recognition Modes by X-Ray, NMR Spectroscopy, and Molecular Modeling. Chem.-A Eur. J..

[25] Vacca A., Francesconi O., Roelens S. (2012). *BC*_50_: A Generalized, Unifying Affinity Descriptor. Chem. Rec..

[26] Nativi C., Francesconi O., Gabrielli G., de Simone I., Turchetti B., Mello T., Mannelli L.D.C., Ghelardini C., Buzzini P., Roelens S. (2012). Aminopyrrolic Synthetic Receptors for Monosaccharides: A Class of Carbohydrate-Binding Agents Endowed with Antibiotic Activity versus Pathogenic Yeasts. Chem.-A Eur. J..

[27] Francesconi O., Nativi C., Gabrielli G., De Simone I., Noppen S., Balzarini J., Liekens S., Roelens S. (2015). Antiviral Activity of Synthetic Aminopyrrolic Carbohydrate Binding Agents: Targeting the Glycans of Viral Gp120 to Inhibit HIV Entry. Chem.-A Eur. J..

[28] Park S.-H., Choi Y.P., Park J., Share A., Francesconi O., Nativi C., Namkung W., Sessler J.L., Roelens S., Shin I. (2015). Synthetic Aminopyrrolic Receptors Have Apoptosis Inducing Activity. Chem. Sci..

[29] Sritharan S., Sivalingam N. (2021). A Comprehensive Review on Time-Tested Anticancer Drug Doxorubicin. Life Sci..

[30] Di Francesco M., Celia C., Cristiano M.C., d’Avanzo N., Ruozi B., Mircioiu C., Cosco D., di Marzio L., Fresta M. (2021). Doxorubicin Hydrochloride-Loaded Nonionic Surfactant Vesicles to Treat Metastatic and Non-Metastatic Breast Cancer. ACS Omega.

[31] Maswadeh H.M., Aljarbou A.N., Alorainy M.S., Rahmani A.H., Khan M.A. (2015). Coadministration of doxorubicin and etoposide loaded in camel milk phospholipids liposomes showed increased antitumor activity in a murine model. Int. J. Nanomed..

[32] Khan M.A., Aljarbou A.N., Aldebasi Y.H., Alorainy M.S., Khan A. (2015). Combination of glycosphingosomes and liposomal doxorubicin shows increased activity against dimethyl-α-benzanthracene-induced fibrosarcoma in mice. Int. J. Nanomed..

[33] Tavano L., Vivacqua M., Carito V., Muzzalupo R., Caroleo M.C., Nicoletta F. (2013). Doxorubicin Loaded Magneto-Niosomes for Targeted Drug Delivery. Colloids Surf B Biointerfaces.

[34] Minamisakamoto T., Nishiguchi S., Hashimoto K., Ogawara K., Maruyama M., Higaki K. (2021). Sequential Administration of PEG-Span 80 Niosome Enhances Anti-Tumor Effect of Doxorubicin-Containing PEG Liposome. Eur. J. Pharm. Biopharm..

[35] Du Y., Xia L., Jo A., Davis R.M., Bissel P., Ehrich M.F., Kingston D.G.I. (2018). Synthesis and Evaluation of Doxorubicin-Loaded Gold Nanoparticles for Tumor-Targeted Drug Delivery. Bioconjug. Chem..

[36] Bagheri E., Alibolandi M., Abnous K., Taghdisi S.M., Ramezani M. (2021). Targeted Delivery and Controlled Release of Doxorubicin to Cancer Cells by Smart ATP-Responsive Y-Shaped DNA Structure-Capped Mesoporous Silica Nanoparticles. J. Mater. Chem. B.

[37] Lu J., Zhao W., Huang Y., Liu H., Marquez R., Gibbs R.B., Li J., Venkataramanan R., Xu L., Li S. (2015). Targeted Delivery of Doxorubicin by Folic Acid-Decorated Dual Functional Nanocarrier. Mol. Pharm..

[38] Liu X., Nie H., Zhang Y., Yao Y., Maitikabili A., Qu Y., Shi S., Chen C., Li Y. (2013). Cell Surface-Specific N-Glycan Profiling in Breast Cancer. PLoS ONE.

[39] Rawat P.S., Jaiswal A., Khurana A., Bhatti J.S., Navik U. (2021). Doxorubicin-Induced Cardiotoxicity: An Update on the Molecular Mechanism and Novel Therapeutic Strategies for Effective Management. Biomed. Pharmacother..

[40] Anderson A.A., Goetzen T., Shackelford S.A., Tsank S. (2000). A Convenient One-Step Synthesis of 2-Hydroxy-1,3,5-Benzenetricarbaldehyde. Synth. Commun..

[41] Bragagni M., Mennini N., Ghelardini C., Mura P. (2012). Development and Characterization of Niosomal Formulations of Doxorubicin Aimed at Brain Targeting. J. Pharm. Pharm. Sci..

[42] Ingallina C., Rinaldi F., Bogni A., Ponti J., Passeri D., Reggente M., Rossi M., Kinsner-Ovaskainen A., Mehn D., Rossi F. (2016). Niosomal Approach to Brain Delivery: Development, Characterization and in Vitro Toxicological Studies. Int. J. Pharm..

[43] Aguilar-Castillo B.A., Santos J.L., Luo H., Aguirre-Chagala Y.E., Palacios-Hernández T., Herrera-Alonso M. (2015). Nanoparticle Stability in Biologically Relevant Media: Influence of Polymer Architecture. Soft Matter.

[44] Maestrelli F., González-Rodríguez M.L., Rabasco A.M., Mura P. (2005). Preparation and Characterisation of Liposomes Encapsulating Ketoprofen–Cyclodextrin Complexes for Transdermal Drug Delivery. Int. J. Pharm..

[45] Cinci L., Luceri C., Bigagli E., Carboni I., Paccosi S., Parenti A., Guasti D., Coronnello M. (2016). Development and Characterization of an in Vitro Model of Colorectal Adenocarcinoma with MDR Phenotype. Cancer Med..

[46] Bigagli E., Cinci L., D’Ambrosio M., Nardini P., Portelli F., Colucci R., Lodovici M., Mugelli A., Luceri C. (2021). Hydrochlorothiazide Use and Risk of Nonmelanoma Skin Cancers: A Biological Plausibility Study. Oxid. Med. Cell. Longev..

[47] Bhupinder K., Newton M.J. (2016). Impact of Pluronic F68 vs Tween 80 on fabrication and evaluation of acyclovir SLNs for skin delivery. Recent Patents Drug Deliv. Formul..

[48] Nayak A.S., Chodisetti S., Gadag S., Nayak U.Y., Govindan S., Raval K. (2020). Tailoring Solulan C24 Based Niosomes for Transdermal Delivery of Donepezil: In Vitro Characterization, Evaluation of PH Sensitivity, and Microneedle-Assisted Ex Vivo Permeation Studies. J. Drug Deliv. Sci. Technol..

[49] Kumar G.P., Rajeshwarrao P. (2011). Nonionic Surfactant Vesicular Systems for Effective Drug Delivery—An Overview. Acta Pharm. Sin. B.

[50] Diskaeva E.I., Bazikov I.A., Vecher O.V., Elbekyan K.S., Mecyaceva L.S. (2018). Investigation Ultrasound Influence on the Size of Niosomes Vesicles on the Based of PEG-12 Dimethicone. Res. J. Pharm. Biol. Chem. Sci..

