# Supplementary material for: Niosomes Functionalized with a Synthetic Carbohydrate Binding Agent for Mannose-Targeted Doxorubicin Delivery
Source: Pharmaceutics. 2023 Jan 10;15(1):235. doi: 10.3390/pharmaceutics15010235 (PMC9863333; doi:10.3390/pharmaceutics15010235)
Supplement: Supplementary file 1 [file pharmaceutics-15-00235-s001.zip › pharmaceutics-2026692-supplementary.pdf]

Supplementary Materials

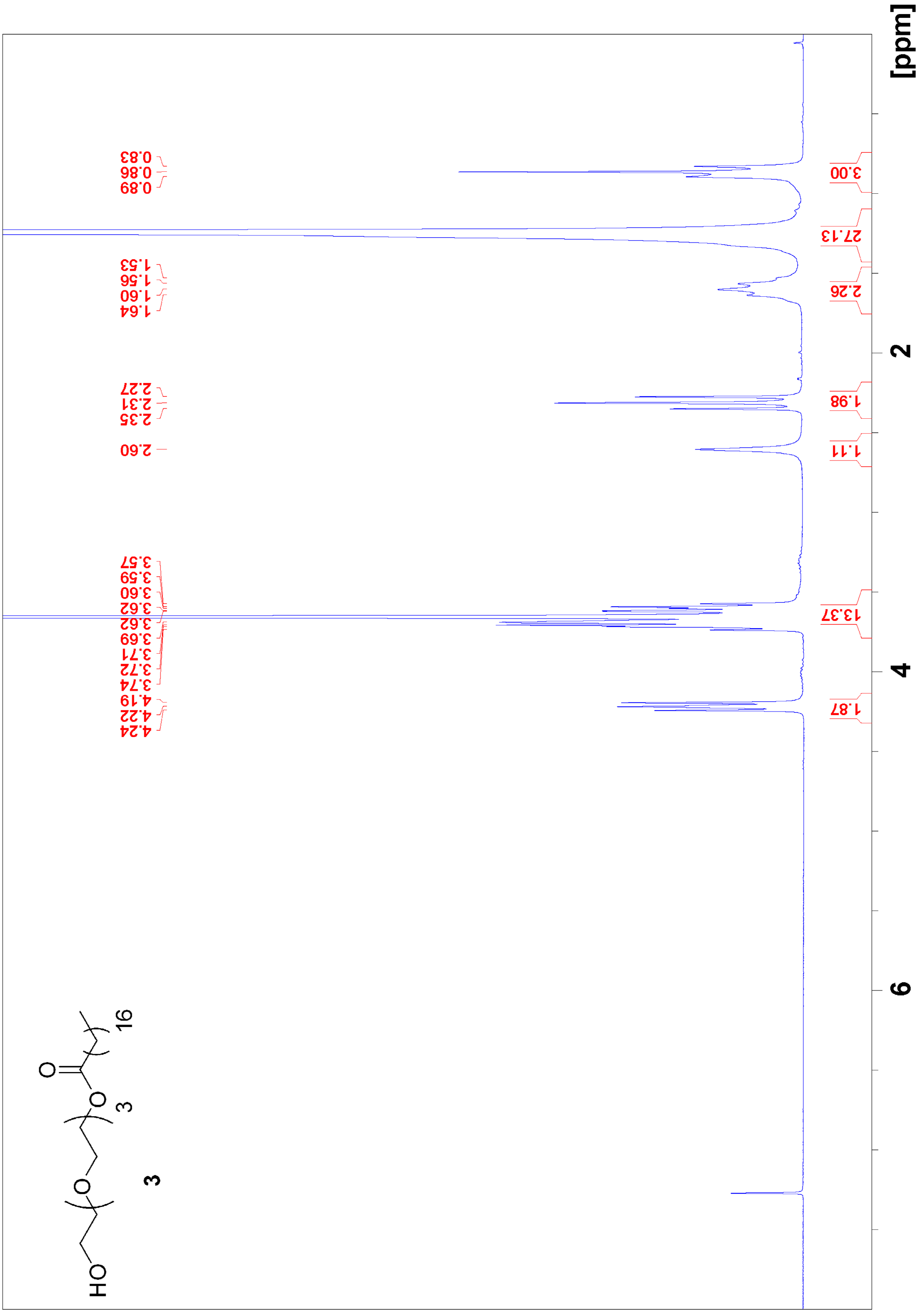

Figure S1. ¹H NMR spectrum of compound **3** (200 MHz, $CDCl_3$).

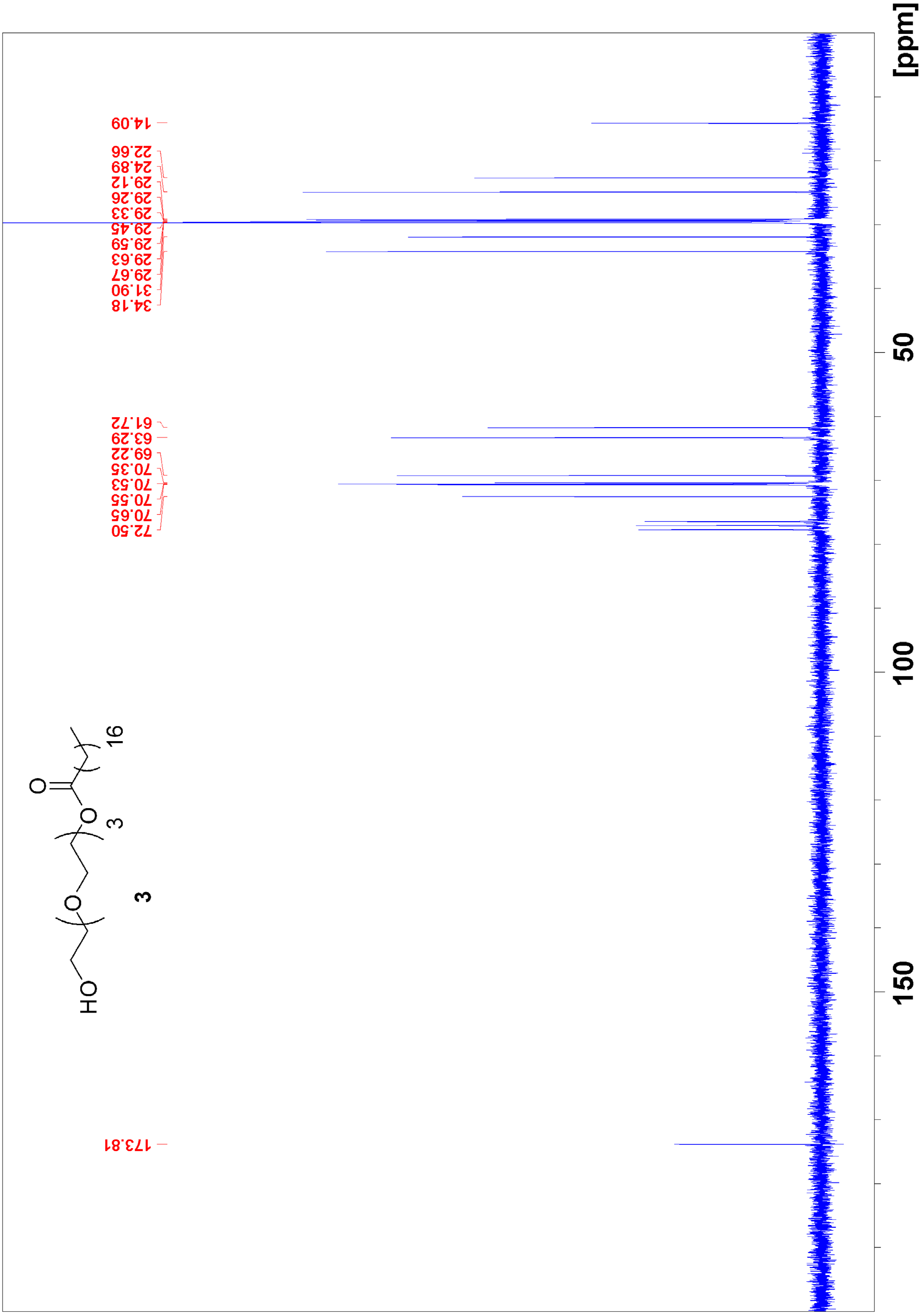

Figure S2. ^{13}C NMR spectrum of compound **3** (50 MHz, $CDCl_3$).

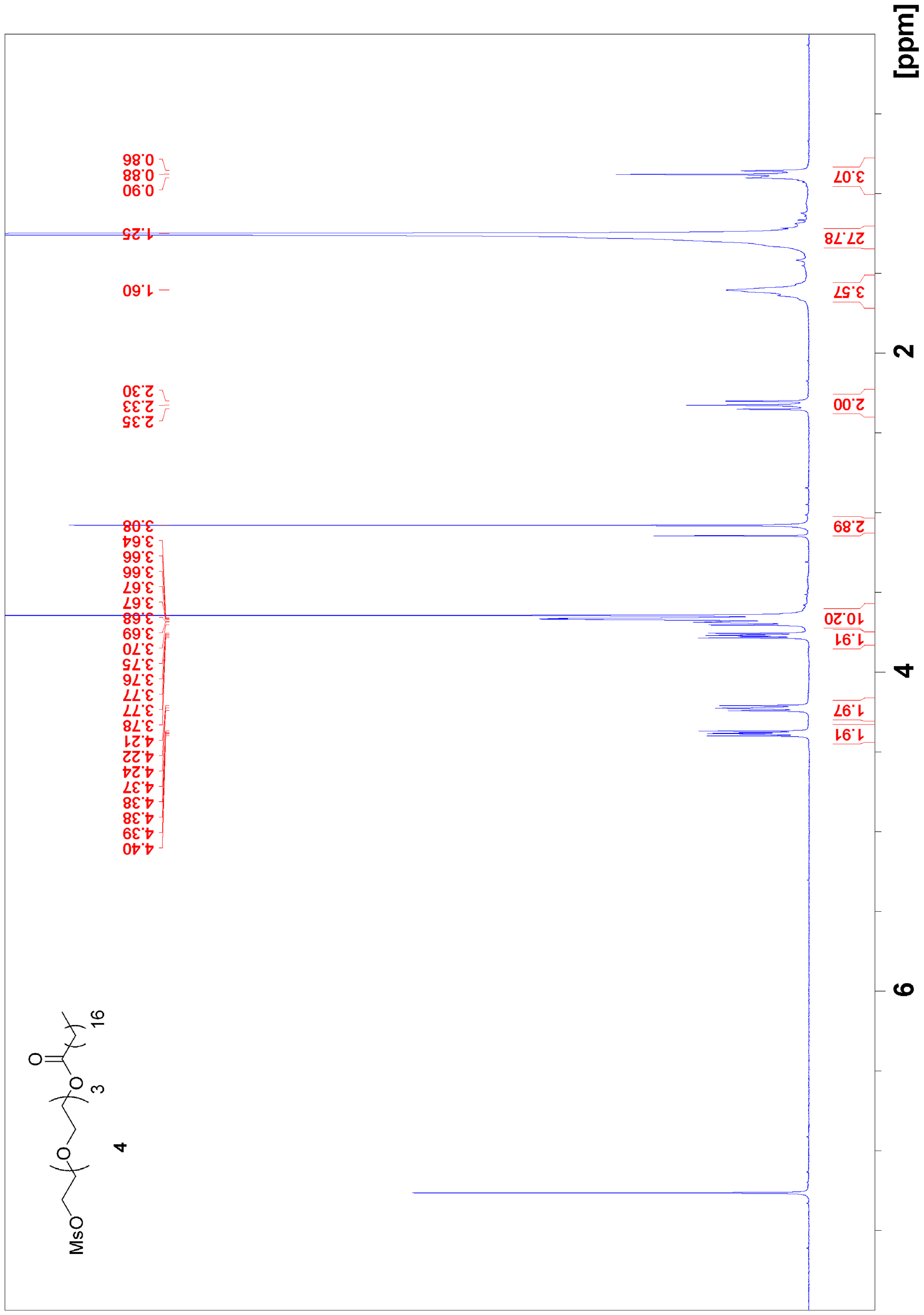

Figure S3. ^{1}H NMR spectrum of compound **4** (300 MHz, $CDCl_3$).

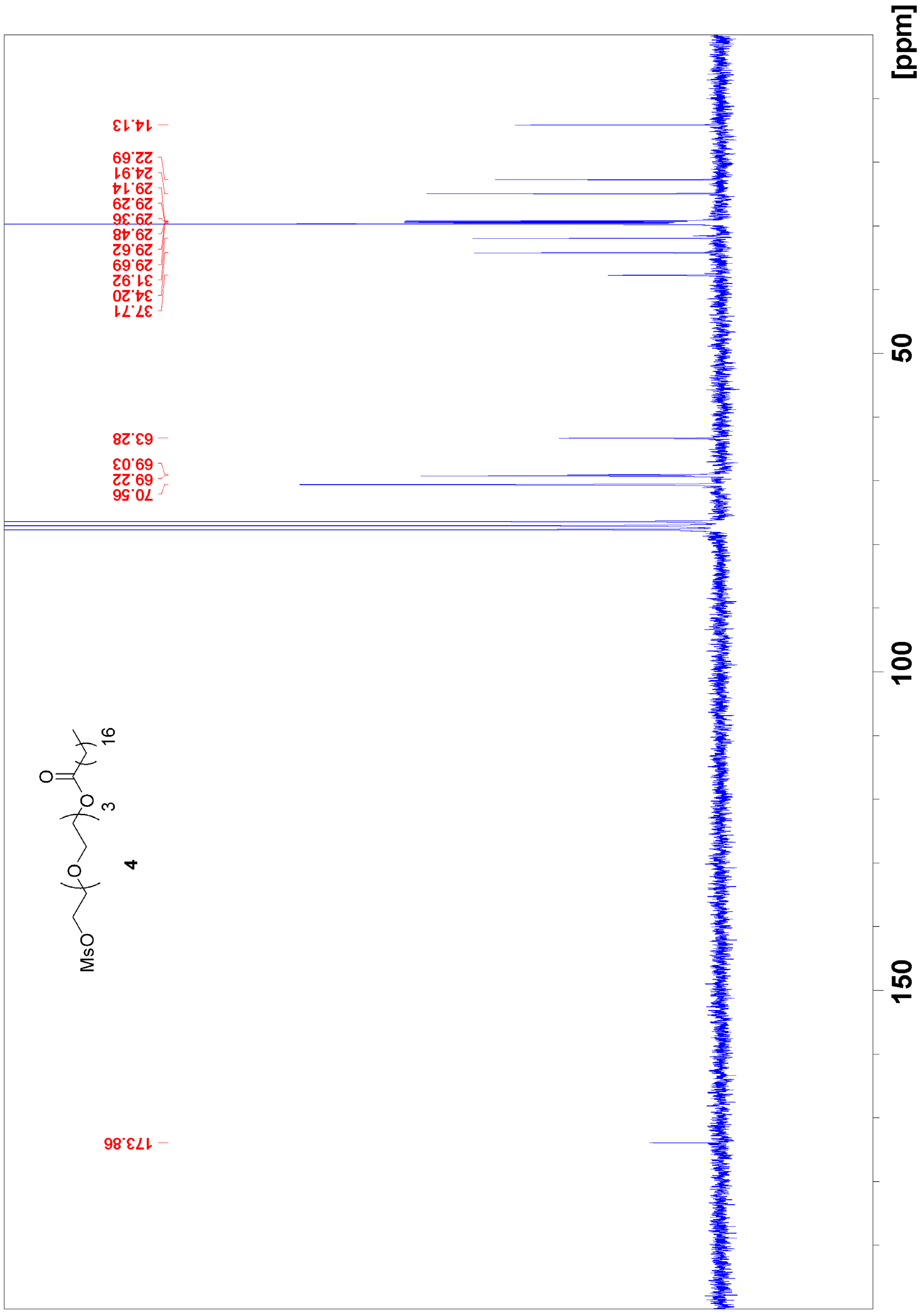

Figure S4. ^{13}C NMR spectrum of compound **4** (50 MHz, $CDCl_3$).

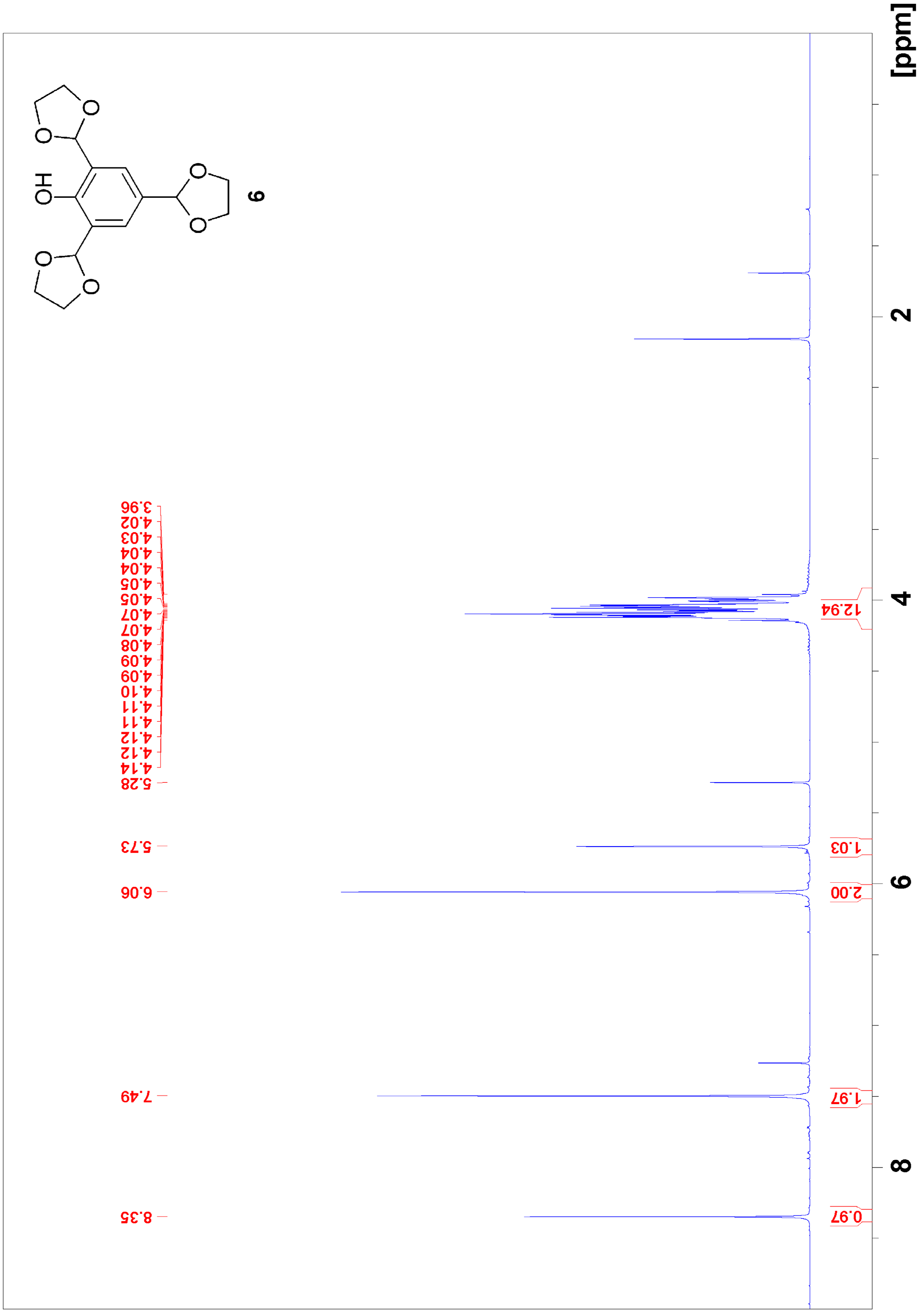

Figure S5. ^{1}H NMR spectrum of compound **6** (200 MHz, $CDCl_3$).

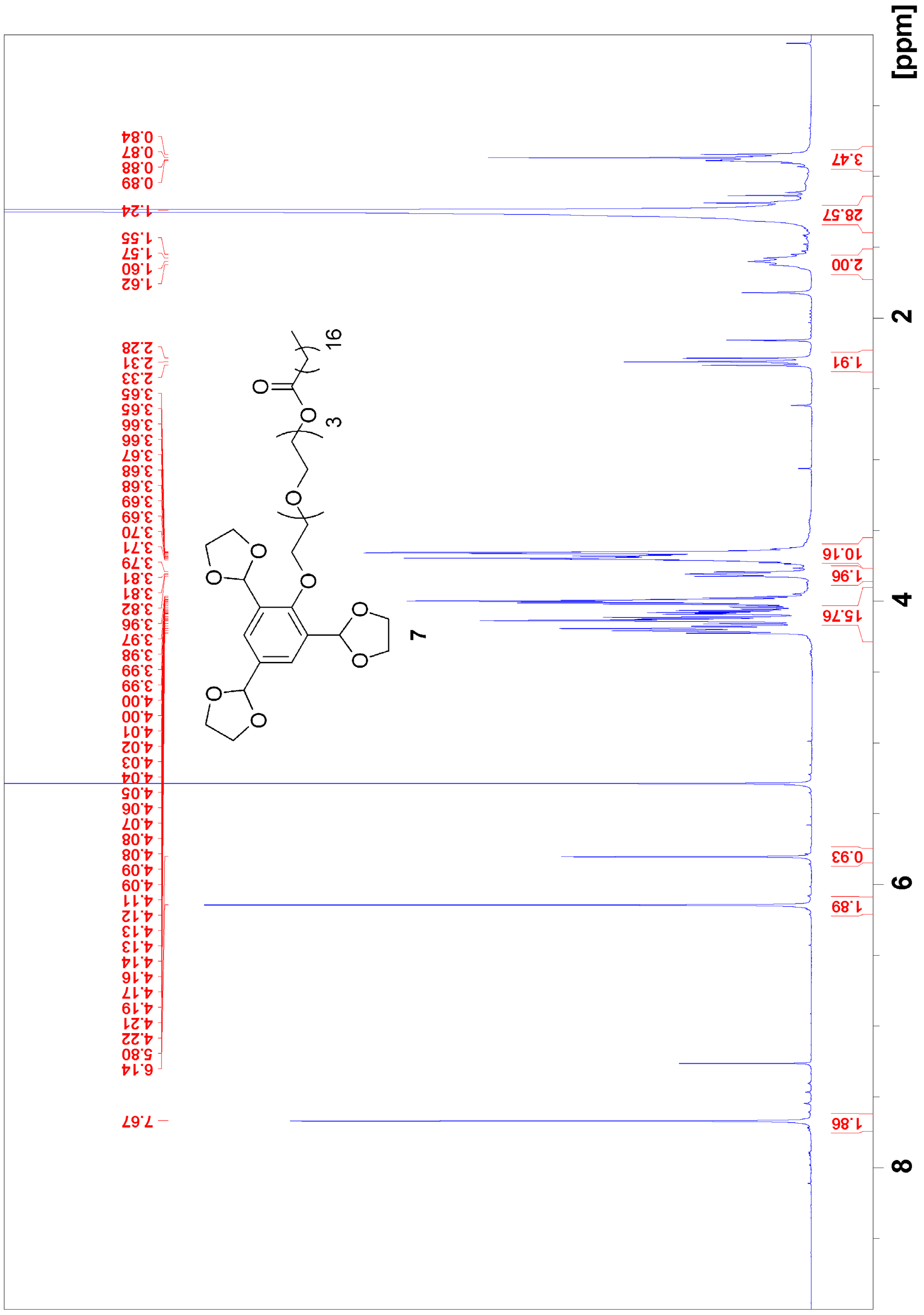

Figure S6. ^{1}H NMR spectrum of compound **7** (300 MHz, $CDCl_3$).

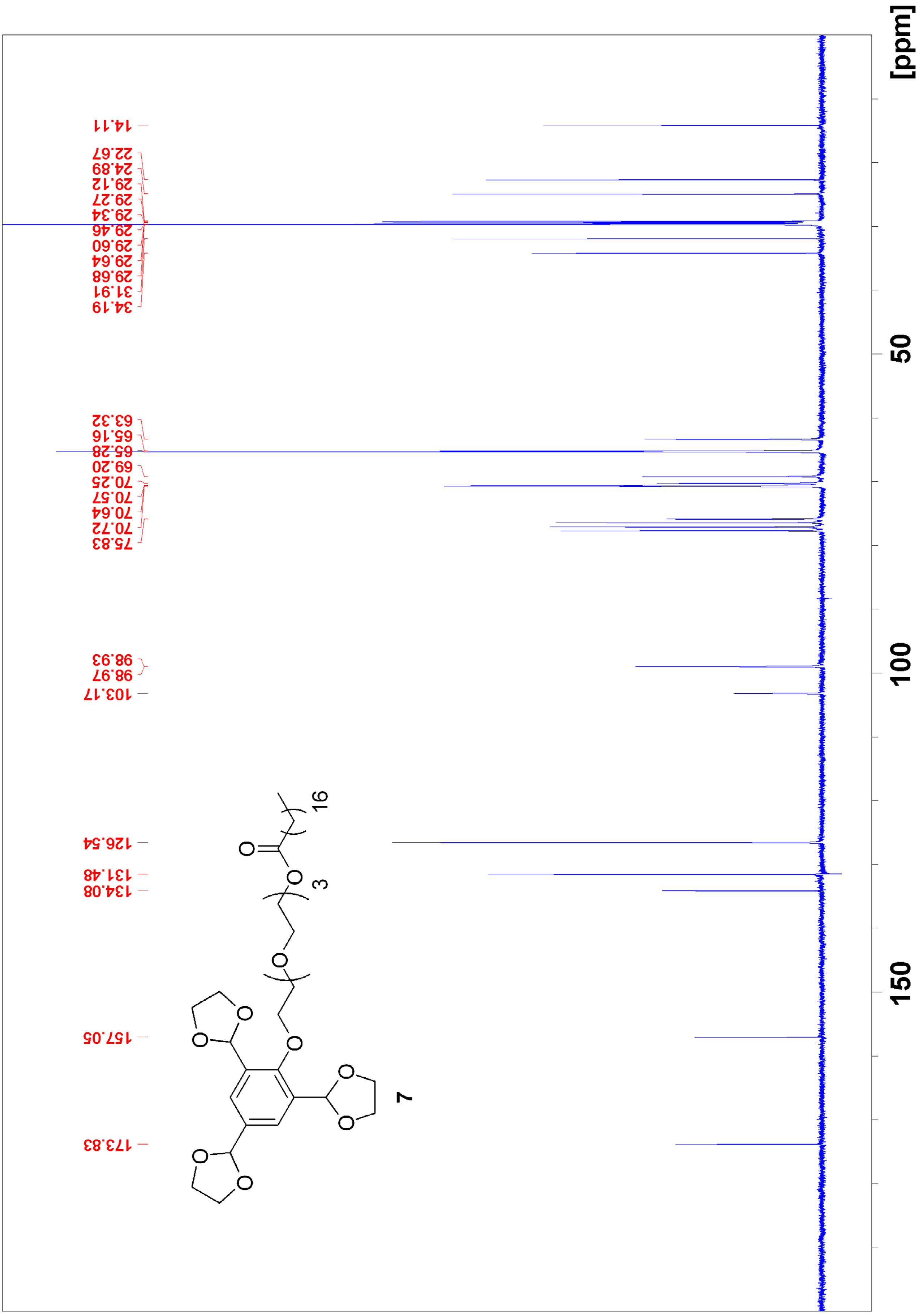

Figure S7. ^{13}C NMR spectrum of compound **7** (50 MHz, $CDCl_3$).

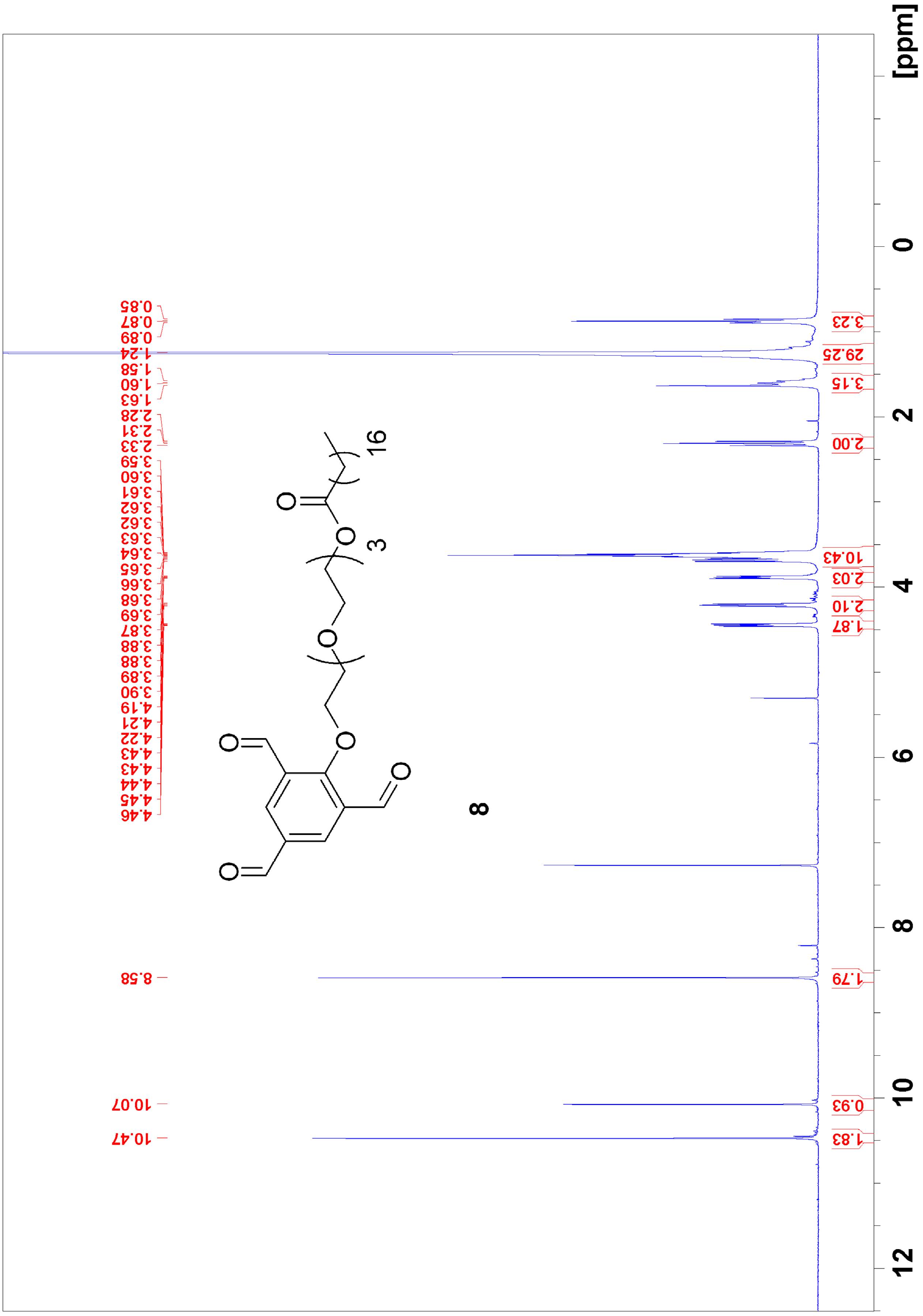

Figure S8. ^{1}H NMR spectrum of compound **8** (300 MHz, $CDCl_3$).

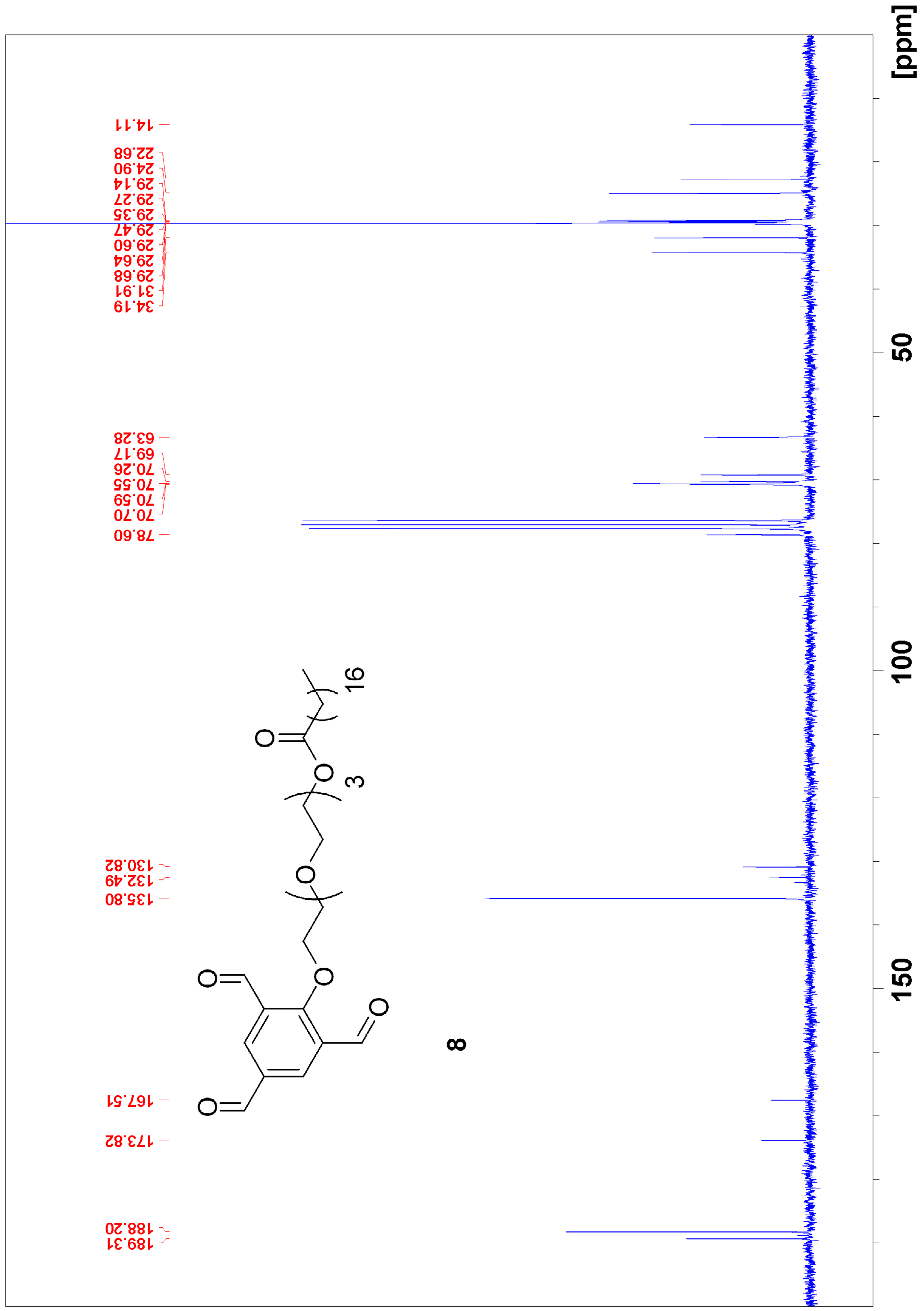

Figure S9. ^{13}C NMR spectrum of compound **8** (50 MHz, $CDCl_3$).

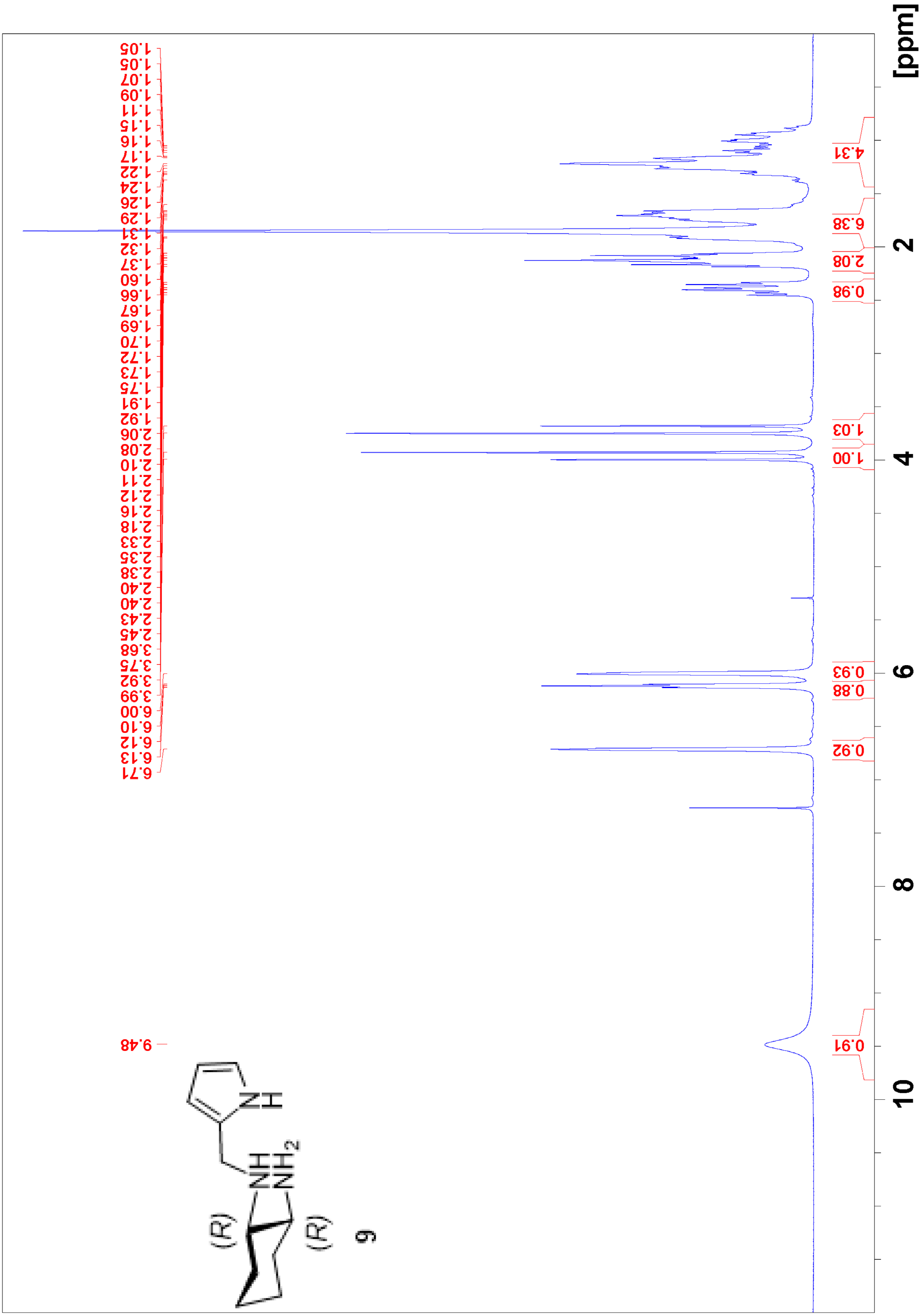

Figure S10. ^{1}H NMR spectrum of compound **9** (200 MHz, $CDCl_3$).

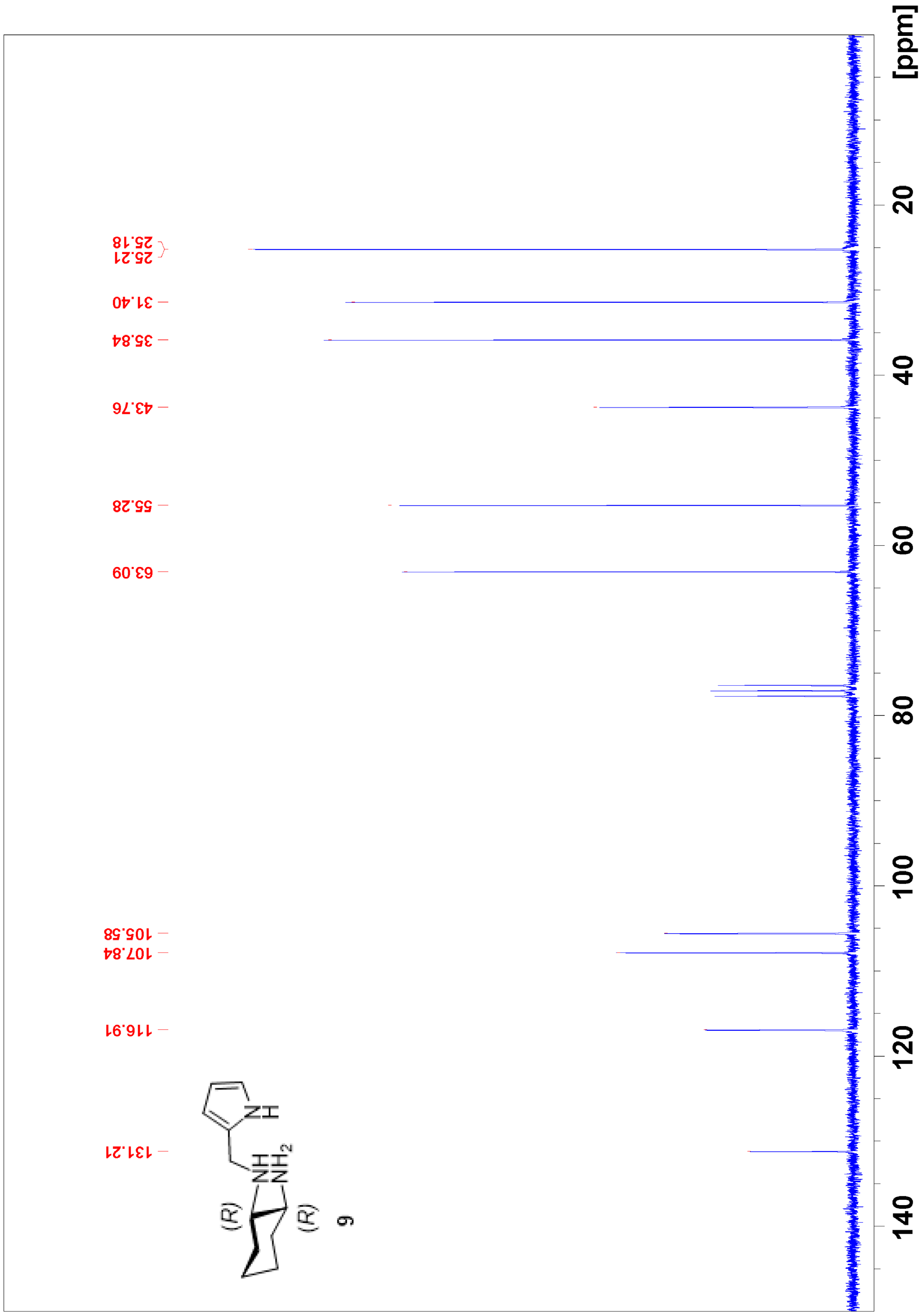

Figure S11. ^{13}C NMR spectrum of compound **9** (50 MHz, $CDCl_3$).

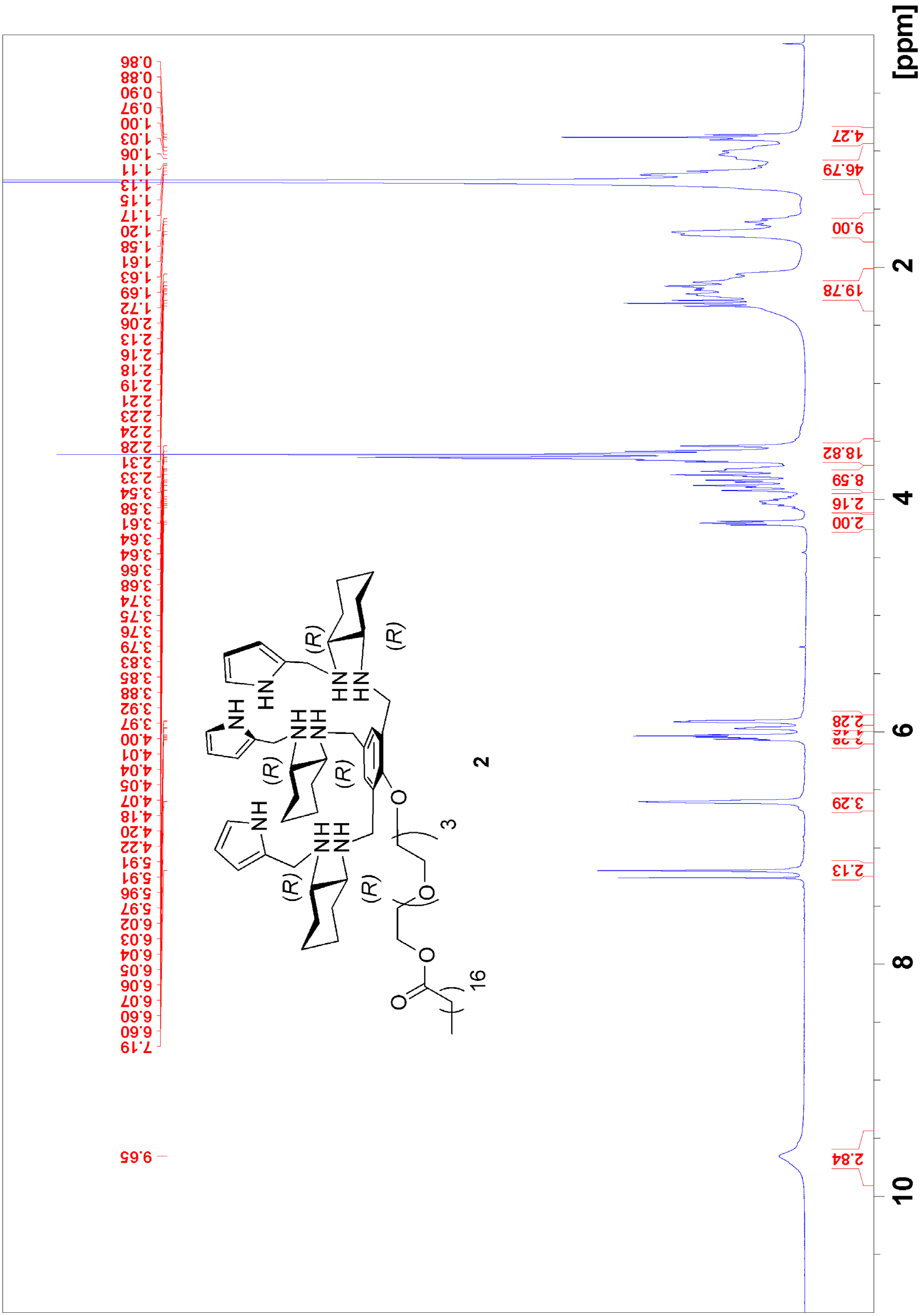

Figure S12. ^{1}H NMR spectrum of receptor **2** (300 MHz, $CDCl_3$).

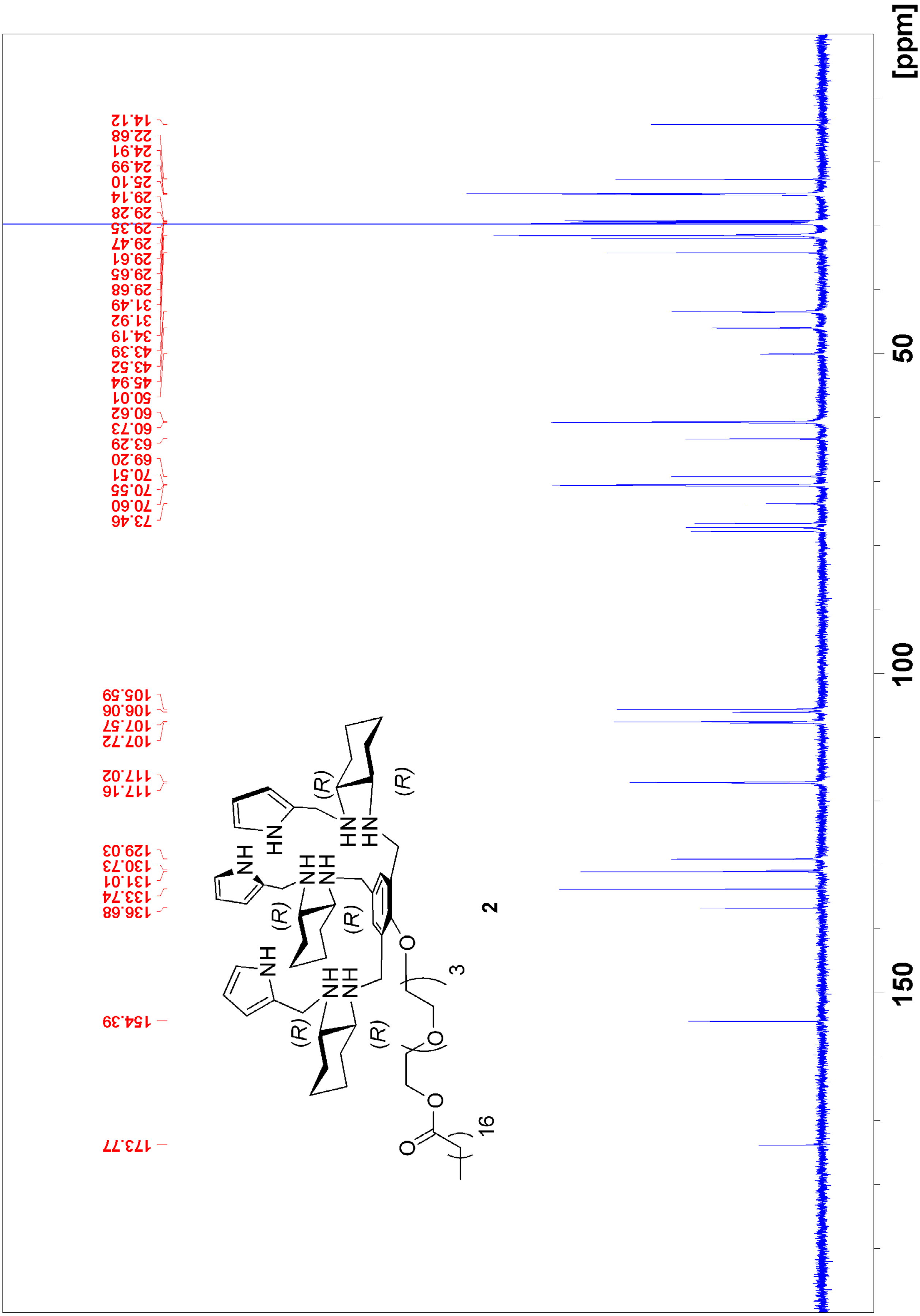

Figure S13. ^{13}C NMR spectrum of receptor **2** (75 MHz, $CDCl_3$).

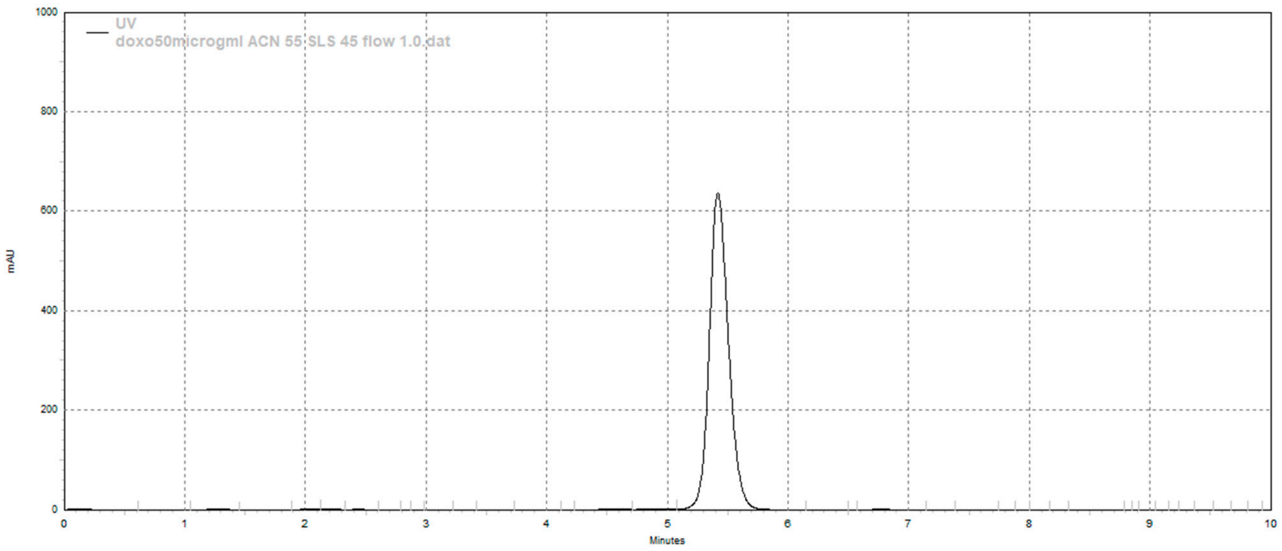

Figure S14. HPLC chromatogram of DOXO.
